# Supplementary material for: Structural basis for recruitment of peptidoglycan endopeptidase MepS by lipoprotein NlpI
Source: Nat Commun. 2024 Jun 27;15:5461. doi: 10.1038/s41467-024-49552-y (PMC11211486; doi:10.1038/s41467-024-49552-y)
Supplement: Supplementary file 1 — Supplementary Information [file 41467_2024_49552_MOESM1_ESM.pdf]

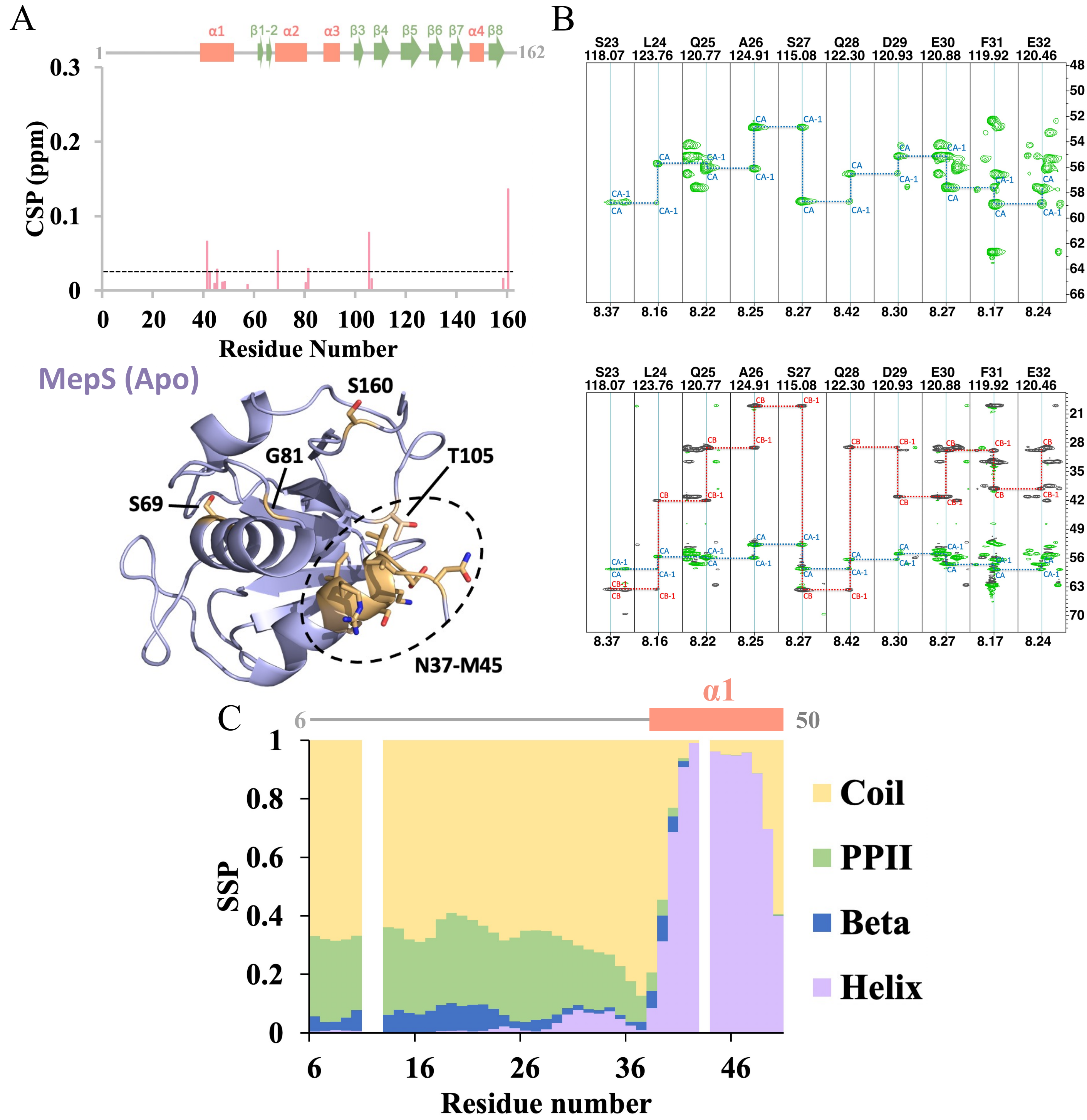

Supplementary Figure 1. NMR analysis of the intrinsically disordered N-terminal of mMepS. (A) Chemical shift perturbation (CSP) analysis was conducted between mMepS and dN36-MepS. CSPs were determined by comparing the peaks in full-length mMepS with those in truncated dN36-MepS. The dashed line (2σ interval) highlights substantial CSP changes (>0.028 ppm). Residue numbers corresponding to the CSP plots are annotated below. Residues exhibiting dramatic CSP alterations are labeled and depicted in a wheat color on the solution structure of MepS (PDB ID 2K1G). (B) Strip plots of HNCA and HNCACB spectra illustrate sequential assignments spanning from Ser23 to Glu32 in mMepS. Intra- and inter-residual correlations of mMepS residues were derived from a combination of three-dimensional HNCA and HNCACB spectra. Positive and negative signal intensities are denoted by green and black colors, respectively. Sequential connectivity is represented by blue and red dashed lines. Residue numbers are labeled alongside corresponding Cα and Cβ resonances. (C) Secondary structure propensity (SSP) analysis of the N-terminal of mMepS was performed using C_{α} and C_{β} chemical shifts. SSPs are visualized through stacked bar plots, with colors representing the relative propensity at each residue. Coils are represented in yellow, PPIIs in green, β-sheets in blue, and α-helices in lavender, as described in the legend. The secondary structural elements of mMepS from residues 6-50 are illustrated above. The source data is included in a separate Source Data file.

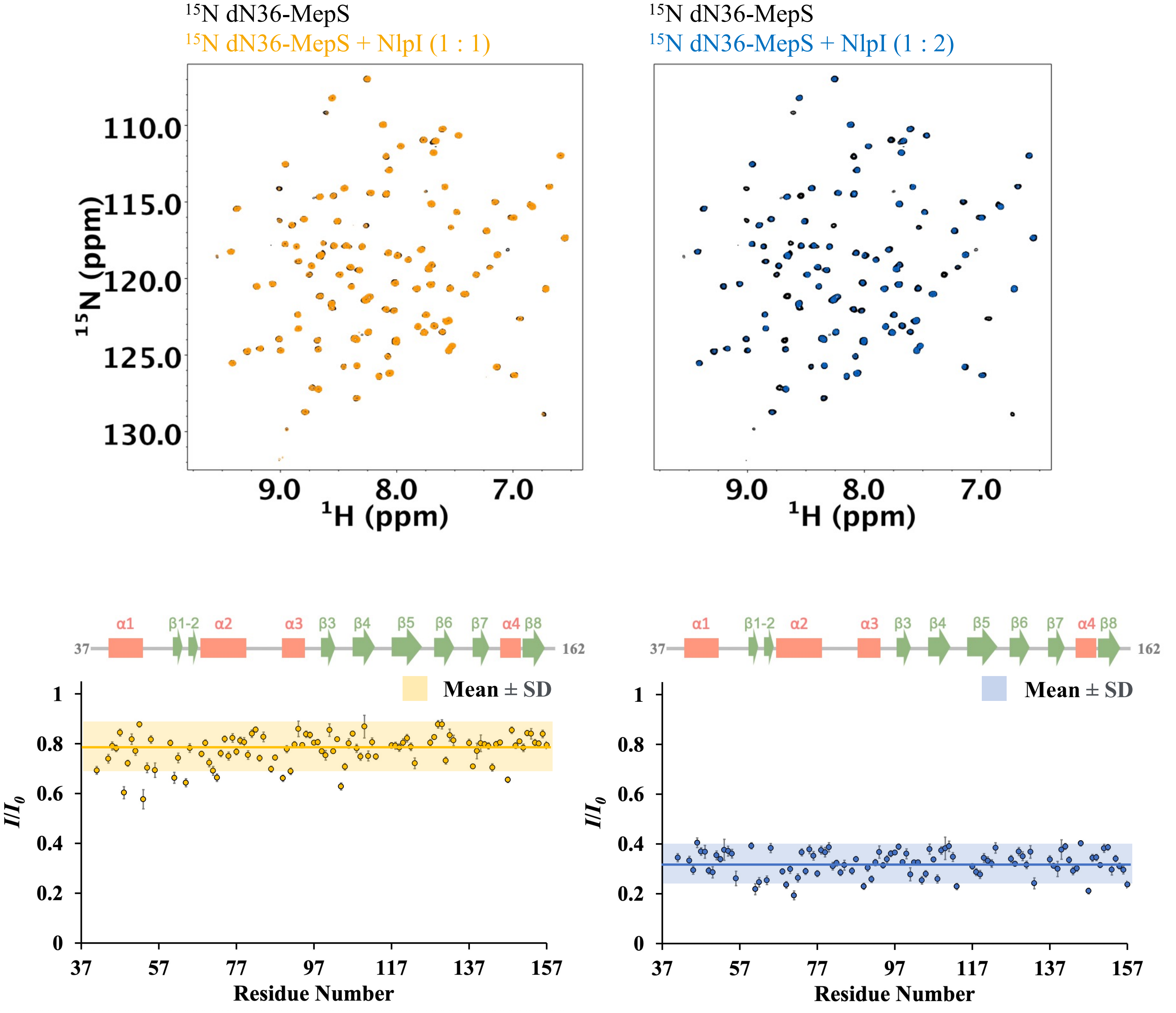

Supplementary Figure 2. NMR titrations of dN36-MepS with NlpI. (Top) The ^{1}H-^{15}N TROSY-HSQC spectra of dN36-MepS alone were superimposed at a protein concentration of 50 μM (depicted in black). Subsequent titrations with NlpI dimer to concentrations of 25 μM (depicted in yellow) and 50 μM (depicted in blue) are also shown. (Bottom) The ratio of signal intensities (I/I_0) for NlpI-bound and free dN36-MepS was determined using residue-resolved NMR. A bar chart depicting the ratio of cross-peak intensities for dN36-MepS upon addition of 25 μM (shown in yellow) and 50 μM NlpI dimer (shown in blue) is presented, while the ranges of mean values ± one standard deviation are depicted with shading. The chart displays residue number versus intensity ratio, with secondary structural elements of dN36-MepS depicted above. All intensity ratios are derived using the NMRView program. Data of one representative experiment performed on $n = 2$ biologically independent samples. Error bars for these ratios are incorporated according to the SD of signal-to-noise ratio observed in the respective spectra. The residues with no intensity ratios may suggest the presence of proline residues and other unassigned residues. Additionally, the absence of intensity ratios could indicate that the signal intensity of these residues is insufficient or that they are overlapping, thereby affecting the accuracy of measurements. The source data is included in a separate Source Data file.

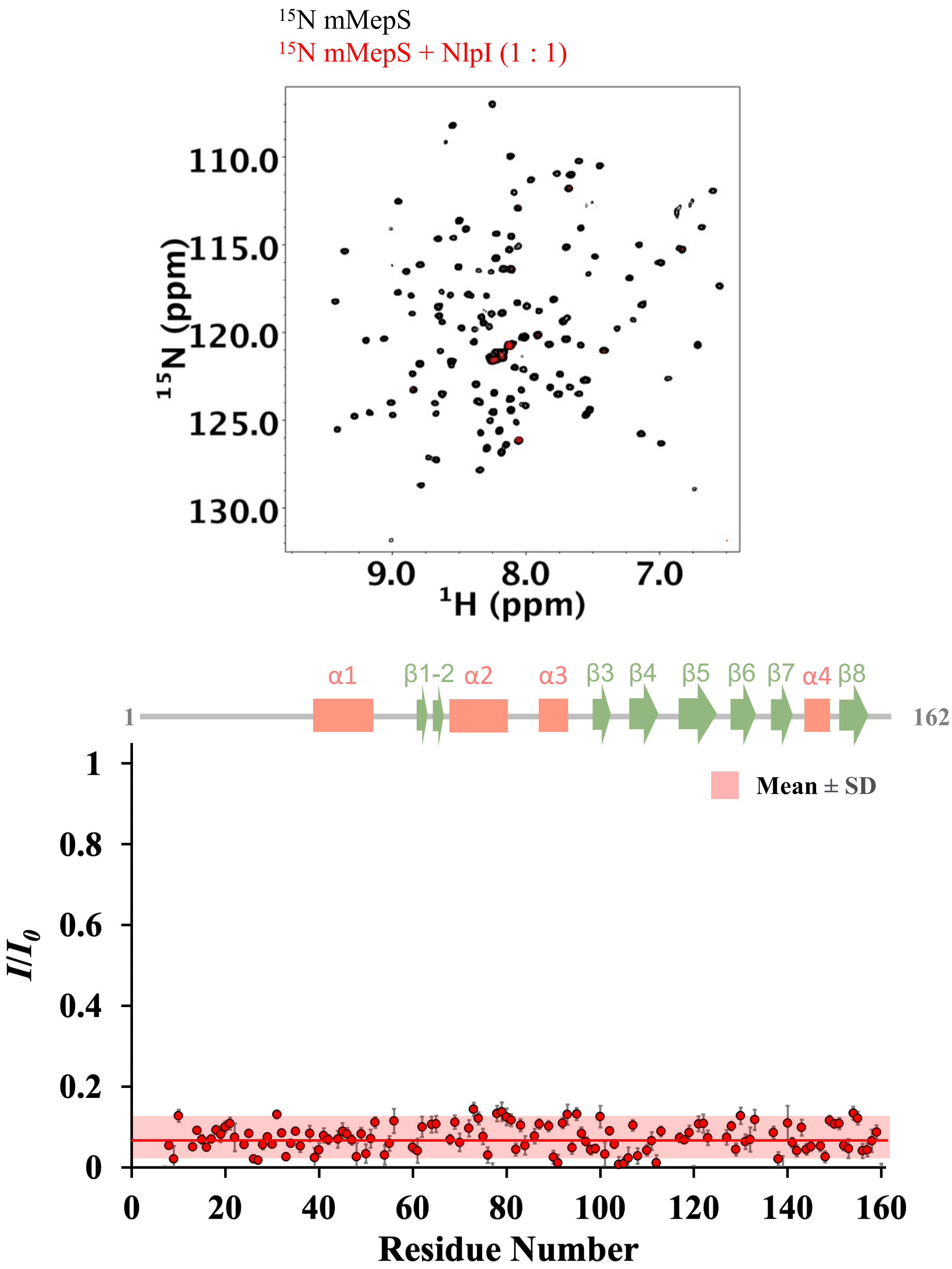

Supplementary Figure 3. NMR titrations of mMepS with NlpI. (Top) The ^{1}H-^{15}N TROSY-HSQC spectrum of mMepS alone were compared at a protein concentration of 50 μM (shown in black). Subsequent titration with NlpI dimer to a concentration of 25 μM is shown in red. (Bottom) The ratio of signal intensities (I/I_0) for NlpI-bound and free mMepS was determined using residue-resolved NMR. A bar chart depicting the ratio of cross-peak intensities for mMepS upon addition of 25 μM (shown in red) is presented, while the ranges of mean values ± one standard deviation are depicted with shading. The chart displays residue number versus intensity ratio, with secondary structural elements of the apo form of mMepS depicted above. All intensity ratios are derived using the NMRView program. Data of one representative experiment performed on $n = 2$ biologically independent samples. Error bars for these ratios are incorporated according to the SD of signal-to-noise ratio observed in the respective spectra. The residues with no intensity ratios may suggest the presence of proline residues and other unassigned residues. Additionally, the absence of intensity ratios could indicate that the signal intensity of these residues is insufficient or that they are overlapping, thereby affecting the accuracy of measurements. The source data is included in a separate Source Data file.

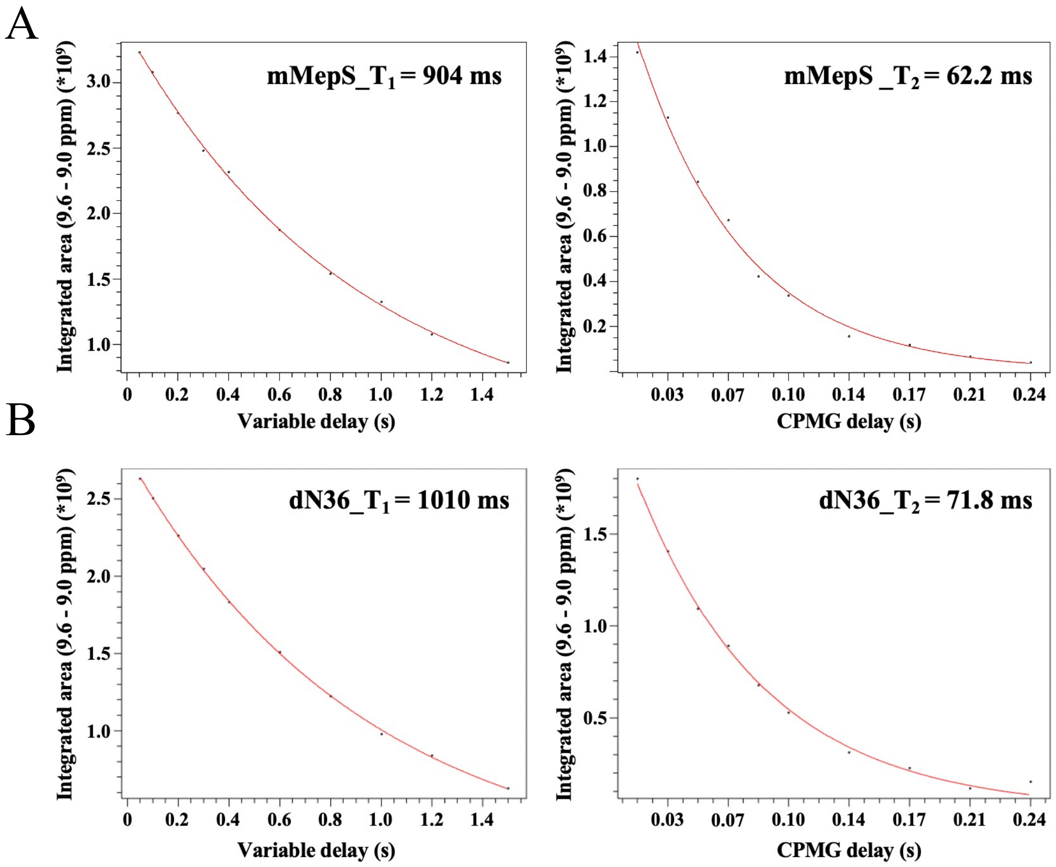

Supplementary Figure 4. ^{15}N T$_1$ and T$_2$ relaxation data for both mMepS and dN36-MepS. (A and B) ^{15}N T_1 and T_2 relaxation data were acquired for mMepS and dN36-MepS using a Bruker AVIII 800 MHz spectrometer at 298 K. Pseudo-2D ^{15}N T_1 and T_2 gradient experiments were employed to collect the data. For the T_1 experiments, spectra were acquired with delays ranging from 20 ms to 1500 ms, with a relaxation delay of 3s. T_2 experiments were conducted with Carr-Purcell-Meiboom-Gill (CPMG) delays ranging from 16 ms to 320 ms, and a relaxation delay of 1.5s. The ^{15}N T_1 and T_2 values were extracted by plotting the decay of integrated ^{1}H intensity between 9.0 – 9.6 ppm and fitting the curves with standard exponential equations. This analysis was performed using the program 't1guide' within Topspin4.0.6 (Bruker). From the obtained data, the rotational correlation time (τ_c) for each protein was calculated using the ^{15}N T_1/T_2 ratio and the approximation of literature relaxation equations:

$$\tau_c = \sqrt{\frac{6T_1}{T_2} - 7} \Big/ 4\,\pi v_N$$

where v_N is the resonance frequency of ^{15}N in Hz. Using this approach, τ_c values of 8.7 and 8.5 ns were obtained for mMepS and dN36-MepS, respectively. Data of one representative experiment performed on $n = 3$ biologically independent samples. The source data is included in a separate Source Data file.

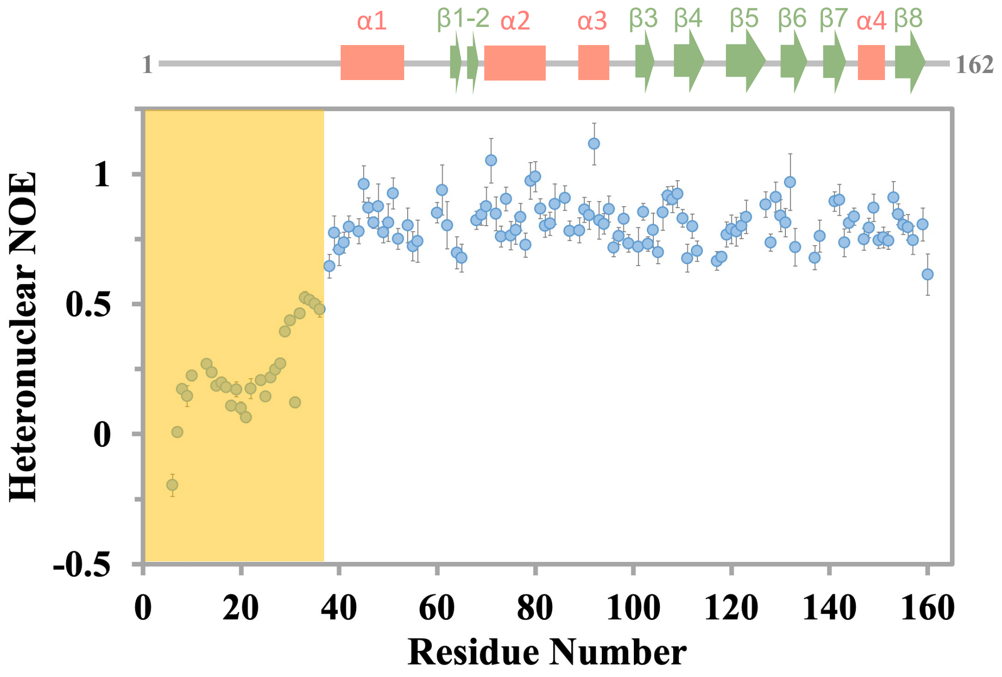

Supplementary Figure 5. Heteronuclear ^{1}H–^{15}N NOEs of mMepS measured at 800 MHz. The heteronuclear NOE values for the peaks of the N-terminal of mMepS exhibit significant reduction, indicating high flexibility on the ps-ns timescale in this region (highlighted in yellow). All heteronuclear NOE values are obtained from the NMRView program. The secondary structural elements of the apo form of mMepS are depicted above. Data of one representative experiment performed on $n = 3$ biologically independent samples. Error bars for these ratios are incorporated according to the SD of signal-to-noise ratio observed in the respective spectra. The residues with no values of NOE may suggest the presence of proline residues and other unassigned residues. Additionally, the absence of intensity ratios could indicate that the signal intensity of these residues is insufficient or that they are overlapping, thereby affecting the accuracy of measurements. The source data is included in a separate Source Data file.

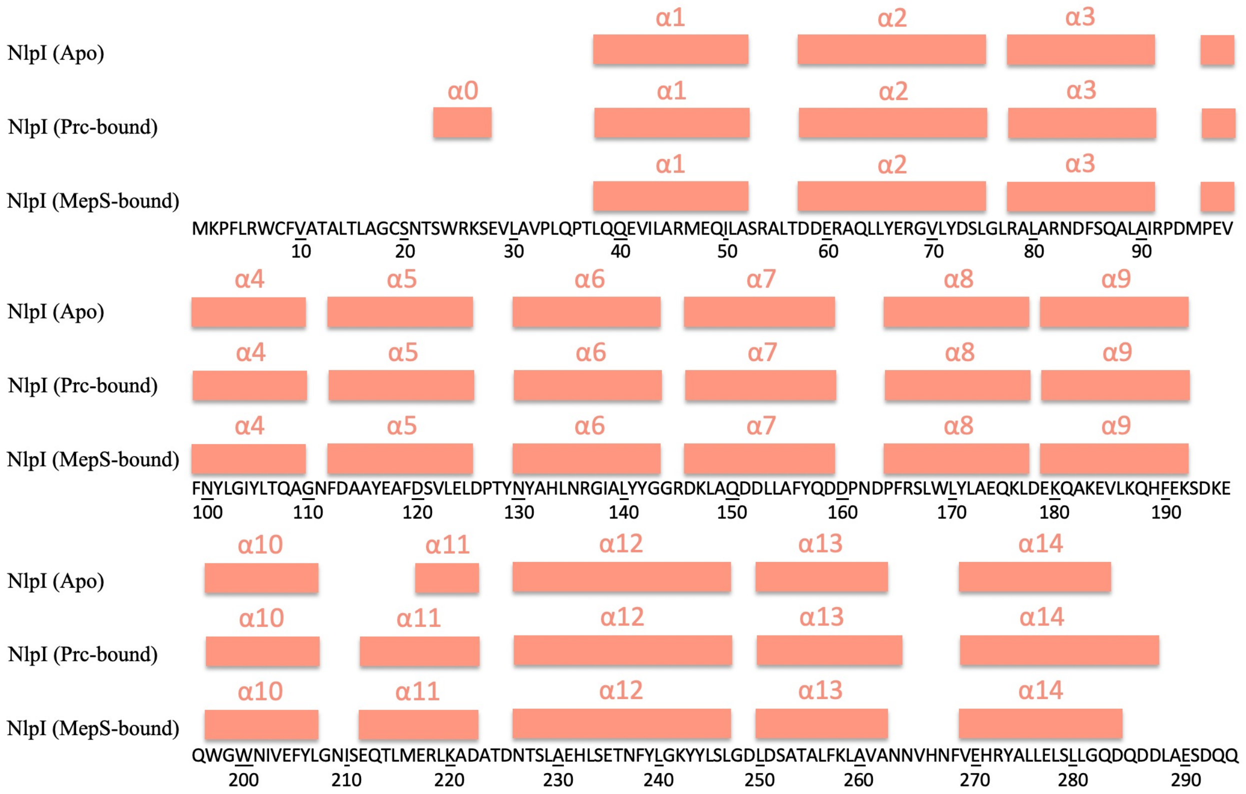

Supplementary Figure 6. Overview of the secondary structure comparison of NlpI in the Apo and bound states. The secondary structure of NlpI in the apo state (PDB ID 1XNF) is depicted in the top row, while the structure of NlpI in Prc-bound (PDB ID 5WQL) and mMepS-bound states (this study) is shown in the center and bottom rows, respectively. These representations are based on atomic resolution protein structures and were generated using the program STRIDE for secondary structure assignment. In these diagrams, red rectangles are used to denote helices.

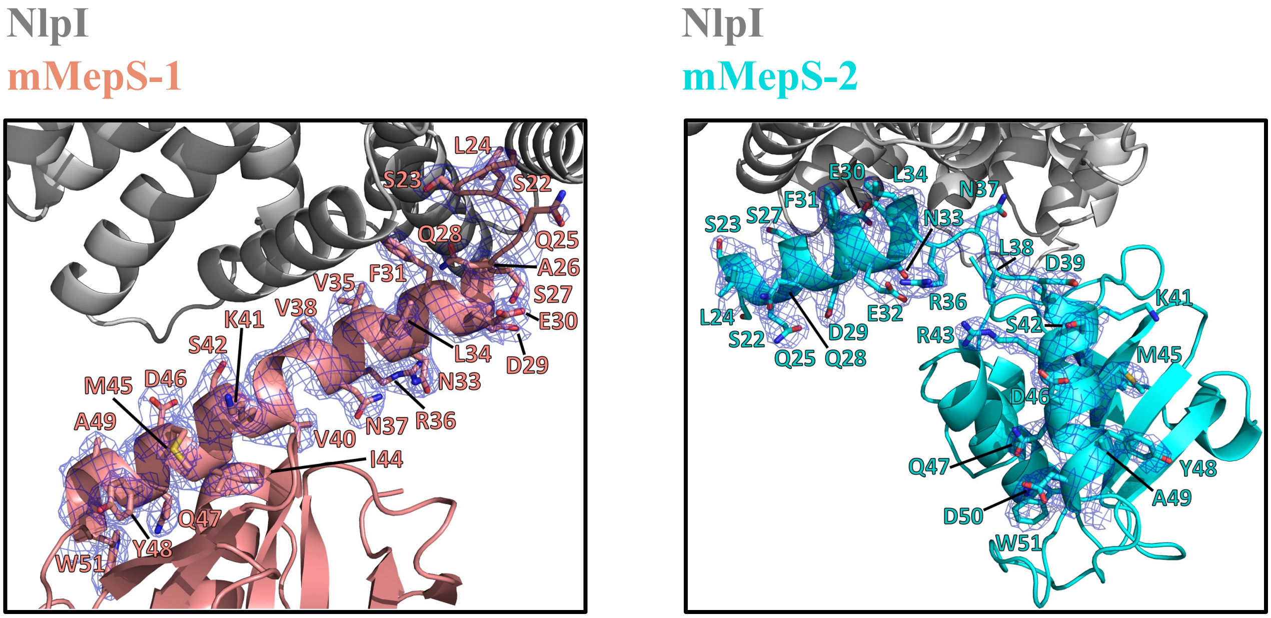

Supplementary Figure 7. Conformational changes in the N-terminal region of mMepS during complex formation with NlpI homodimer. The figures illustrate the two key interaction sites responsible for inducing the coil to helix transition in the N-terminal region of mMepS upon binding with NlpI. In the atomic resolution crystal structure of the NlpI-mMepS complex, NlpI is depicted in gray, mMepS-1 in salmon, and mMepS-2 in cyan. The N-terminal helix region of two mMepS molecules is represented using an omit map at the contour level 1.0 and as a blue mesh, with the N-terminal residues labeled around the side chain.

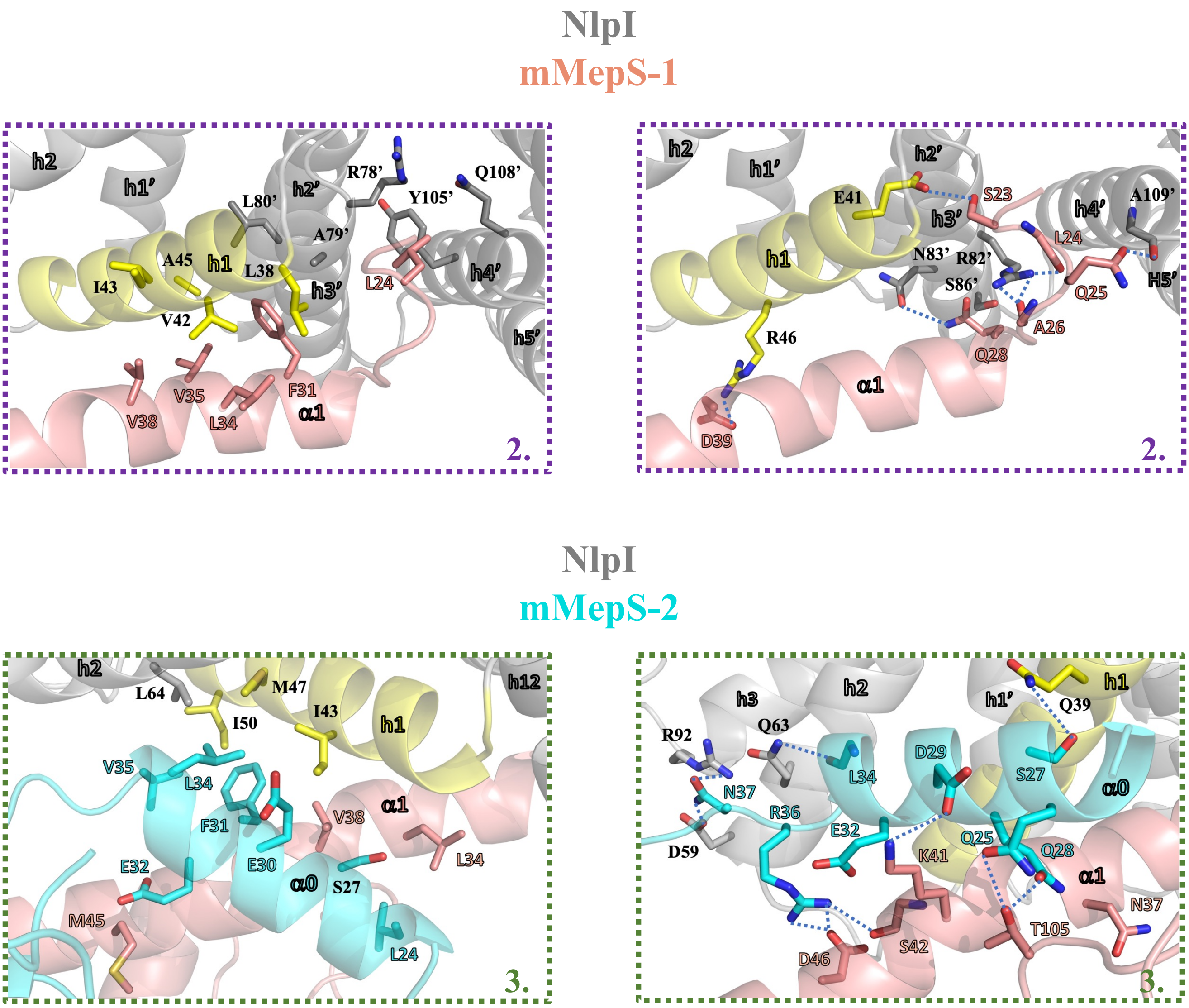

Supplementary Figure 8. Close-up views of interacting residues of mMepS-1, mMepS-2 located at the N-terminal helices with NlpI. Interface 2: The extended α1 region of mMepS-1 is involved in the formation of hydrophobic contacts with specific residues of NlpI h1 and NlpI' h3'-h4', including L38, V42, I43, A45, R78'-L80', Y105', and Q108'. In addition, polar contacts are established between residues S23, L24, Q25, A26, Q28, and D39 of mMepS-1 with residues E41 and R46 of NlpI h1, and residues R82', N83', S86', and A109' of NlpI' h3'-h4’. Interface 3: Residues L24, S27, E30, F31, E32, L34, and V35 of mMepS-2 interact hydrophobically with residues I43, M47, I50, and L64 of NlpI h1-h2, and residues L34, V38, and M45 of mMepS-1 α1. Furthermore, polar contacts are established between residues Q25, S27, Q28, D29, E32, L34, R36, and N37 of mMepS-2 with residues N37, K41, S42, D46, and T105 of mMepS-1, as well as residues Q39, D59, Q63, and R92 of NlpI, suggesting a network of interactions between the proteins. Additionally, the cartoon transparency is set at 50%.

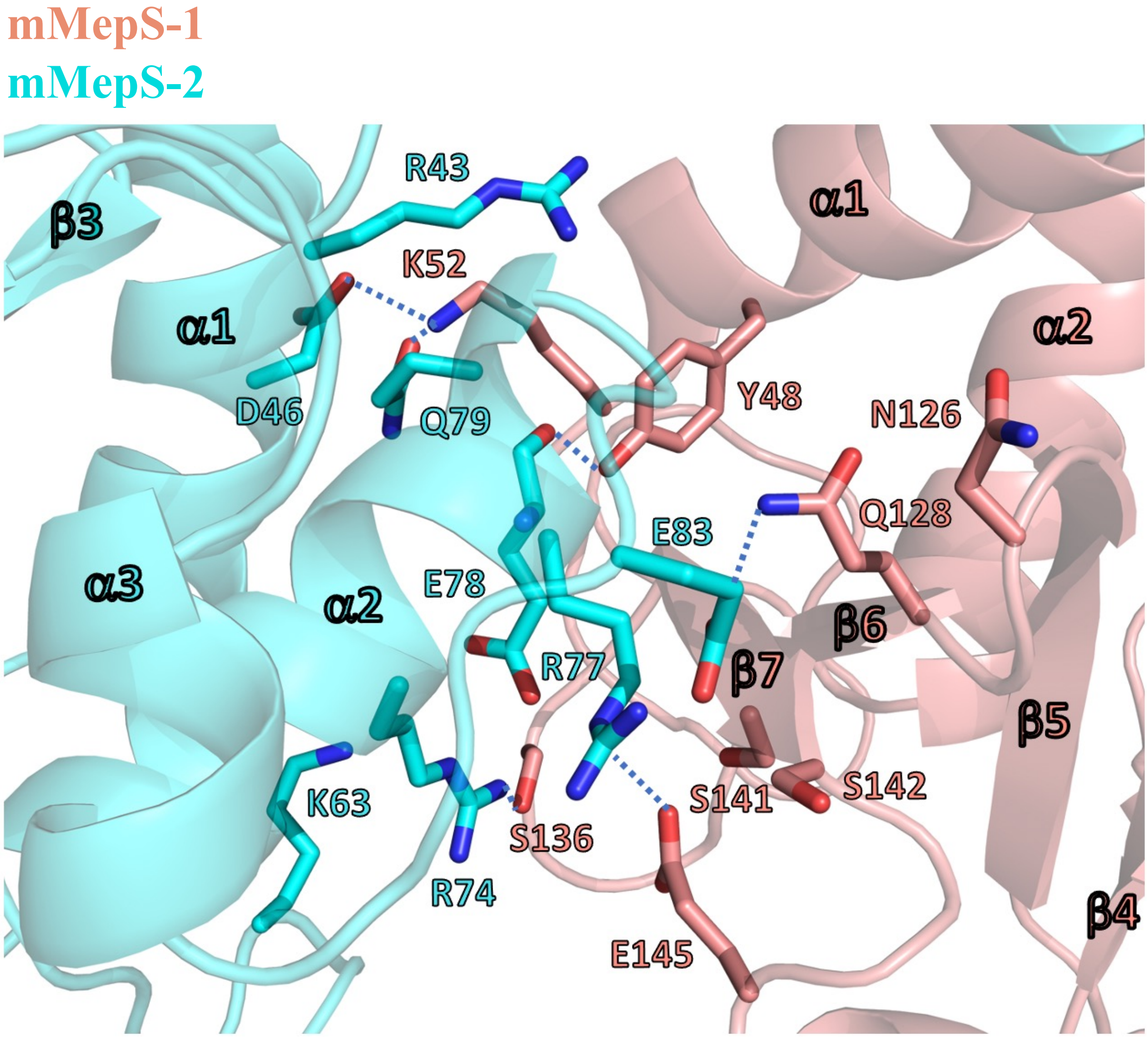

Supplementary Figure 9. Analysis of mMepS dimeric interface at the core structure. The stability of the mMepS dimerization interface is maintained by hydrophilic interactions. Numerous hydrogen bonds and salt bridges have been identified between the core domains of mMepS-1 (Y48, K52, N126, Q128, S136, S141, S142, and E145) and mMepS-2 (R43, D46, K63, R74, R77, E78, Q79 and E83).

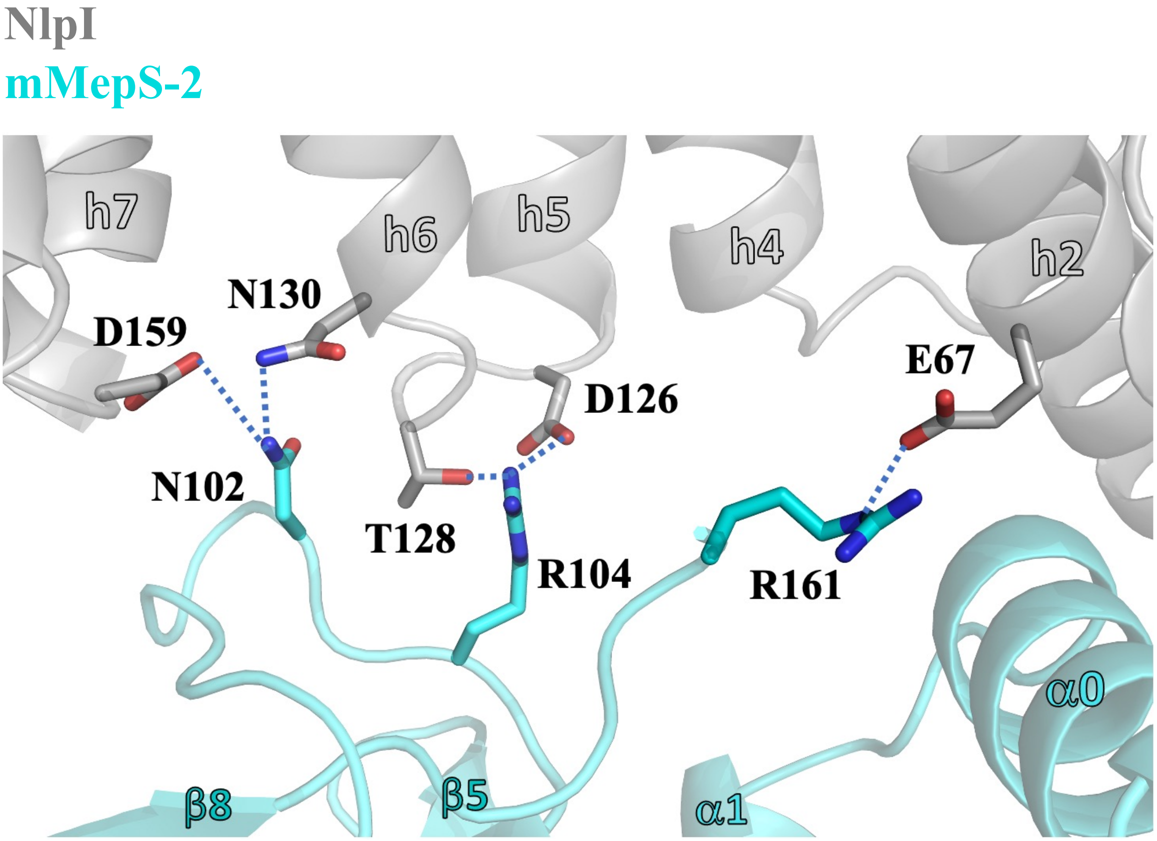

Supplementary Figure 10. A close-up view of interacting residues between mMepS-2 and NlpI. The stability of this interface is maintained through polar interactions involving specific residues. Specifically, residues N102, R104, and R161 of mMepS-2 form interactions with residues E67, D126, N130, and D159 of NlpI.

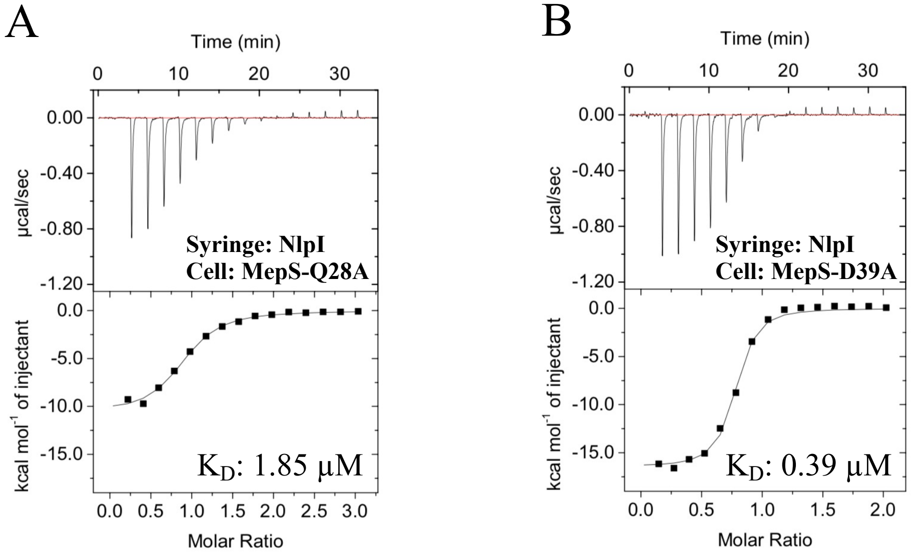

Supplementary Figure 11. ITC-based characterization of MepS mutants interacting with NlpI. (A and B) The interaction of NlpI with MepS-Q28A and MepS-D39A was analyzed using ITC. The raw data after baseline correction (top) and the binding isotherms (bottom) were obtained by plotting the integrated heat peaks against the molar ratio of ligands. The K_D values, derived from fitting to 1:1 binding model, are listed in Table S2.

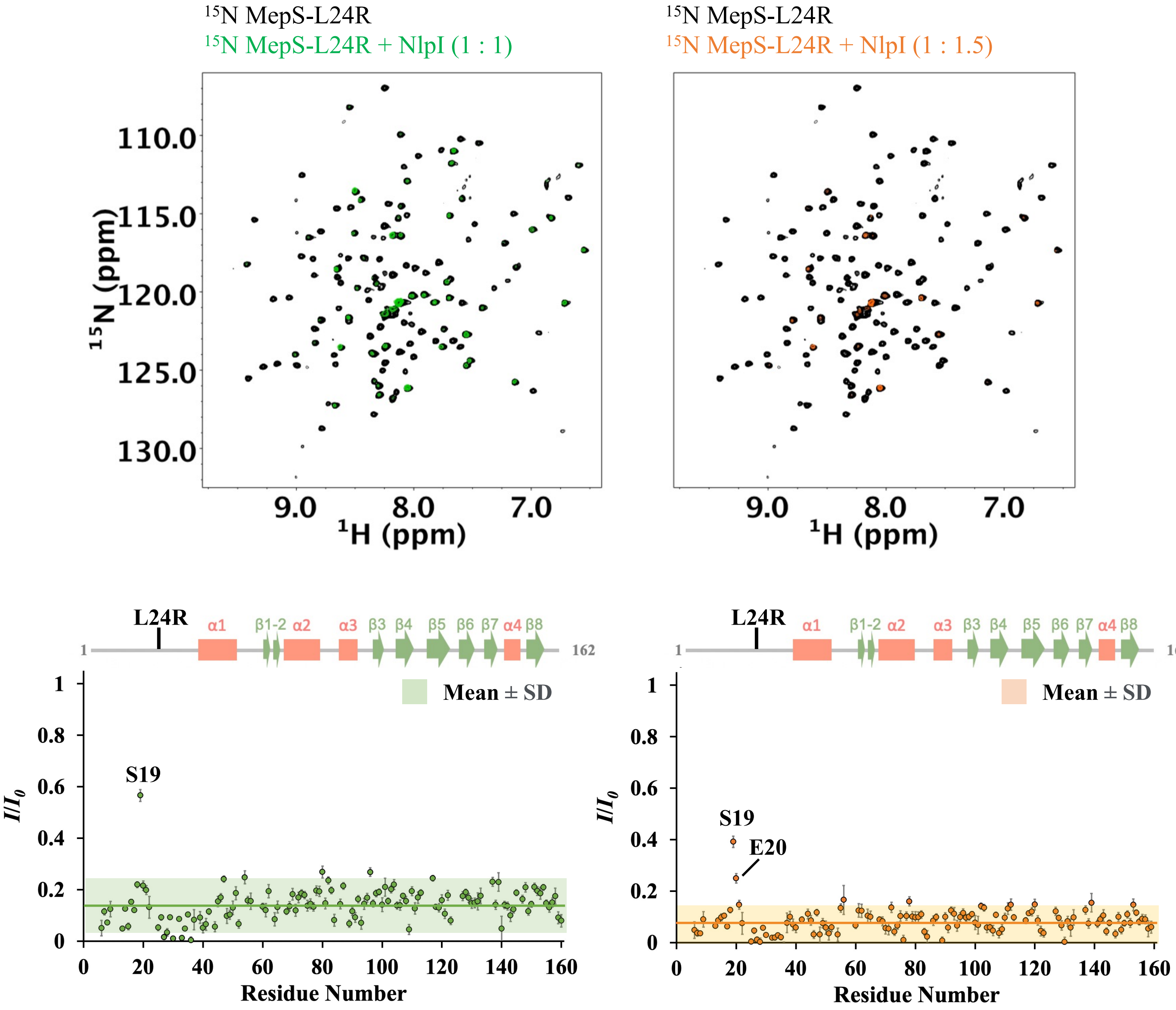

Supplementary Figure 12. NMR titrations of MepS-L24R with NlpI. (Top) The ^{1}H-^{15}N TROSY-HSQC spectra of MepS-L24R alone were superimposed at a protein concentration of 50 μM (depicted in black). Subsequent titrations with NlpI dimer to concentrations of 25 μM (depicted in green) and 37.5 μM (depicted in orange) are also shown. (Bottom) The ratio of signal intensities (I/I_0) for NlpI-bound and free MepS-L24R was determined using residue-resolved NMR. A bar chart depicting the ratio of cross-peak intensities for MepS-L24R upon addition of 25 μM (shown in green) and 37.5 μM NlpI dimer (shown in orange) is presented, while the ranges of mean values ± one standard deviation are depicted with shading. The chart displays residue number versus intensity ratio, with secondary structural elements of the apo form of mMepS depicted above. All intensity ratios are derived using the NMRView program. Data of one representative experiment performed on $n = 2$ biologically independent samples. Error bars for these ratios are incorporated according to the SD of signal-to-noise ratio observed in the respective spectra. The residues with no intensity ratios may suggest the presence of proline residues and other unassigned residues. Additionally, the absence of intensity ratios could indicate that the signal intensity of these residues is insufficient or that they are overlapping, thereby affecting the accuracy of measurements. The source data is included in a separate Source Data file.

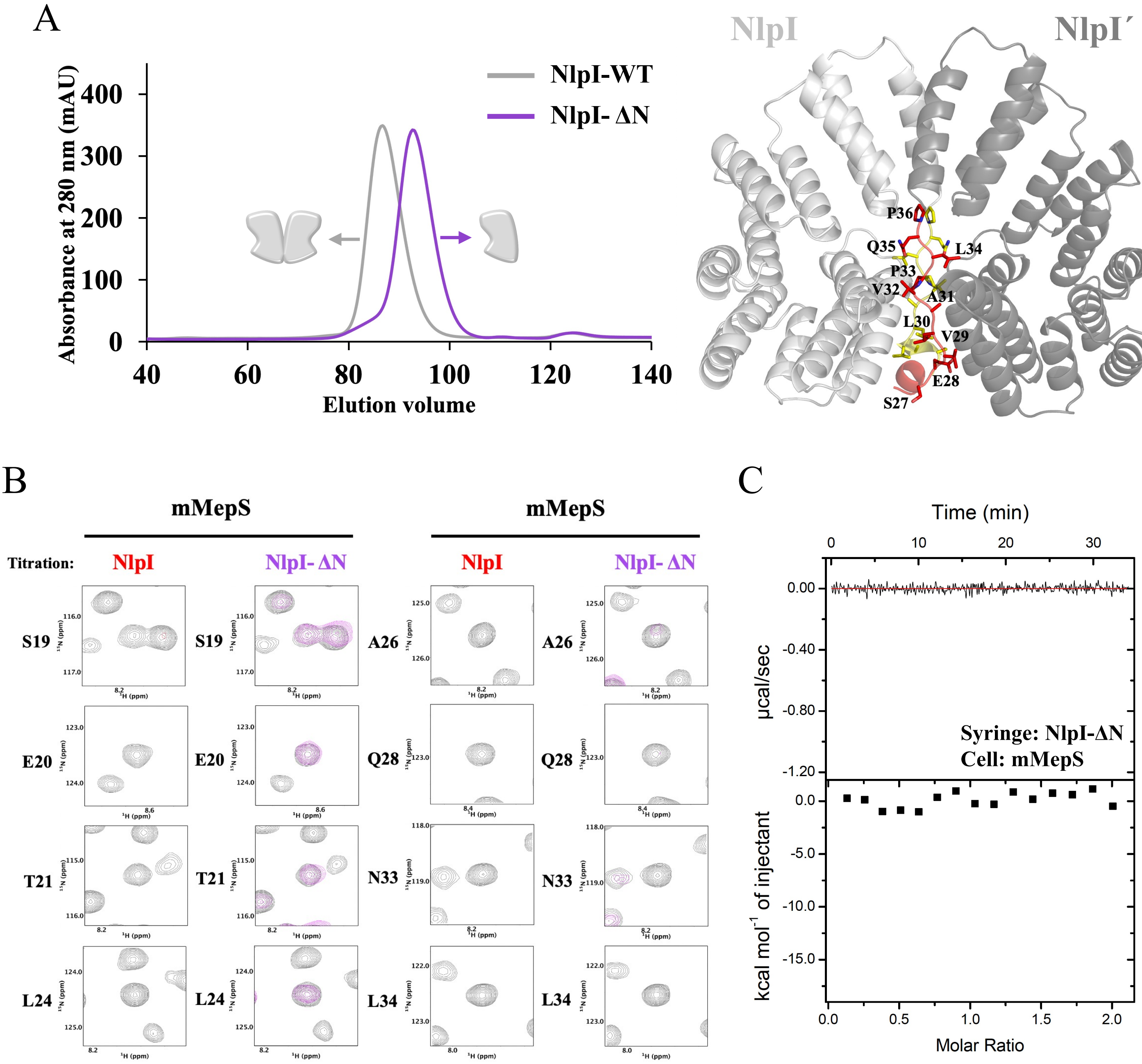

Supplementary Figure 13. Mutational analysis of the stoichiometry of NlpI-mMepS complex and NlpI-mMepS binding interface. (A) Equal amounts of NlpI-WT and NlpI-ΔN proteins were analyzed using gel filtration with a Superdex 200 16/600 GL column. The results revealed that the NlpI-ΔN variant, lacking residues M1-P36, significantly impacted the dimerization of NlpI, suggesting the importance of N-terminal residues in this process. The specific N-terminal residues involved in NlpI dimerization were labeled and highlighted in red and yellow on the NlpI structure (gray cartoon, PDB ID: 1XNF). (B) NMR analysis was conducted to compare the spectra of mMepS when titrated with NlpI or NlpI-ΔN. Overlapped ^{1}H-^{15}N TROSY-HSQC spectra of 50 μM mMepS (black) titrated with 50 μM NlpI (red) or 50 μM NlpI-ΔN dimer (purple) were compared, with specific attention to the labeled N-terminal residues of interest. (C) ITC was employed to analyze the interaction between NlpI-ΔN and mMepS, revealing no binding between them. The source data is included in a separate Source Data file.

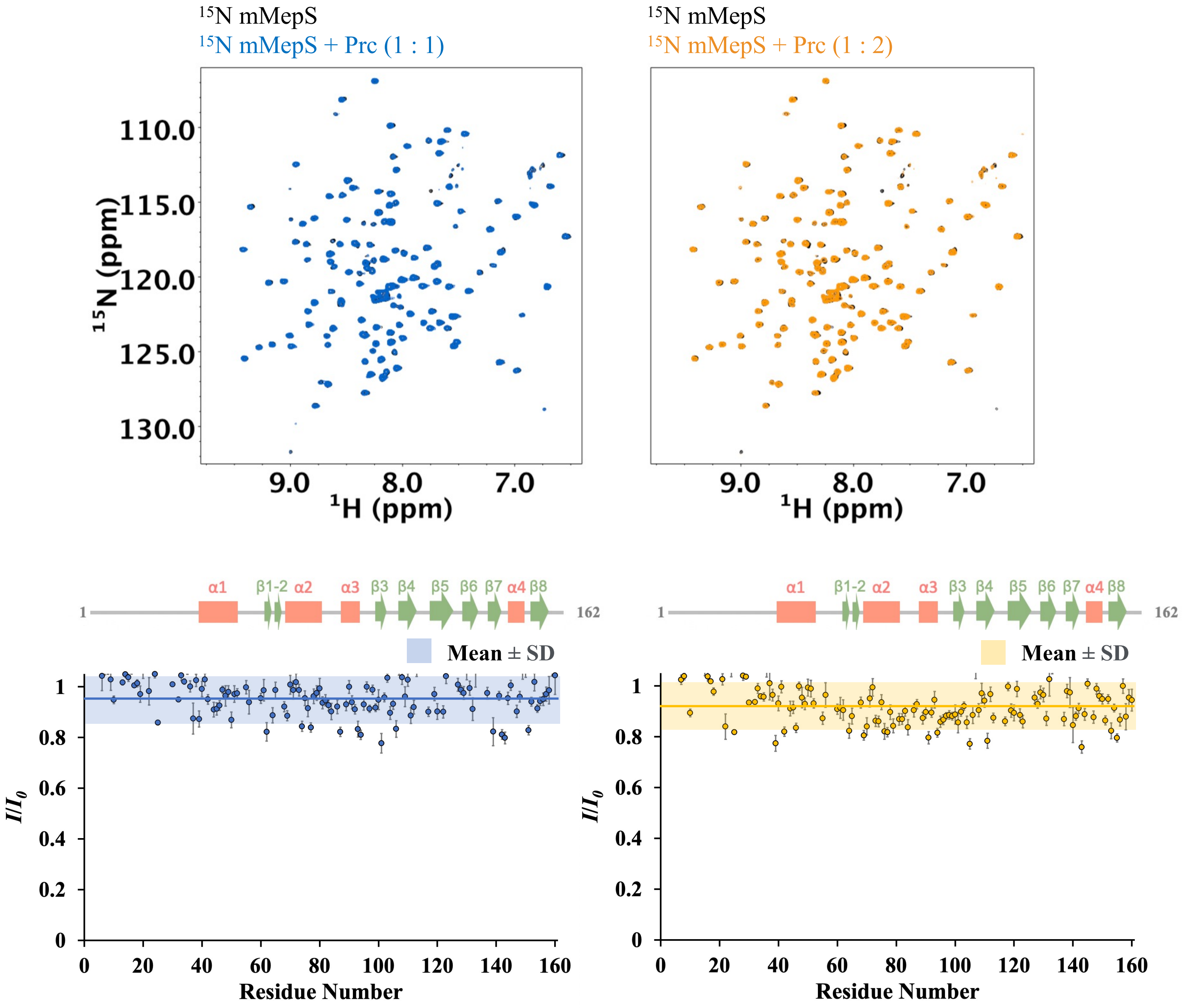

Supplementary Figure 14. NMR titrations of mMepS with Prc^{SK}. (Top) The ^{1}H-^{15}N TROSY-HSQC spectra of mMepS alone were superimposed at a protein concentration of 50 μM (depicted in black). Subsequent titrations with Prc^{SK} to concentrations of 50 μM (depicted in blue) and 100 μM (depicted in yellow) are also shown. (Bottom) The ratio of signal intensities (I/I_0) for Prc^{SK}-titrated and free mMepS was determined using residue-resolved NMR. A bar chart depicting the ratio of cross-peak intensities for mMepS upon addition of 50 μM (shown in blue) and 100 μM Prc^{SK} (shown in yellow) is presented, while the ranges of mean values ± one standard deviation are depicted with shading. The chart displays residue number versus intensity ratio, with secondary structural elements of the apo form of mMepS depicted above. All intensity ratios are derived using the NMRView program. Data of one representative experiment performed on $n = 2$ biologically independent samples. Error bars for these ratios are incorporated according to the SD of signal-to-noise ratio observed in the respective spectra. In these experiments, it was observed that even with a doubling of the amount of Prc, there was no significant change in the signal intensity of mMepS. The residues with no intensity ratios may suggest the presence of proline residues and other unassigned residues. Additionally, the absence of intensity ratios could indicate that the signal intensity of these residues is insufficient or that they are overlapping, thereby affecting the accuracy of measurements. The source data is included in a separate Source Data file.

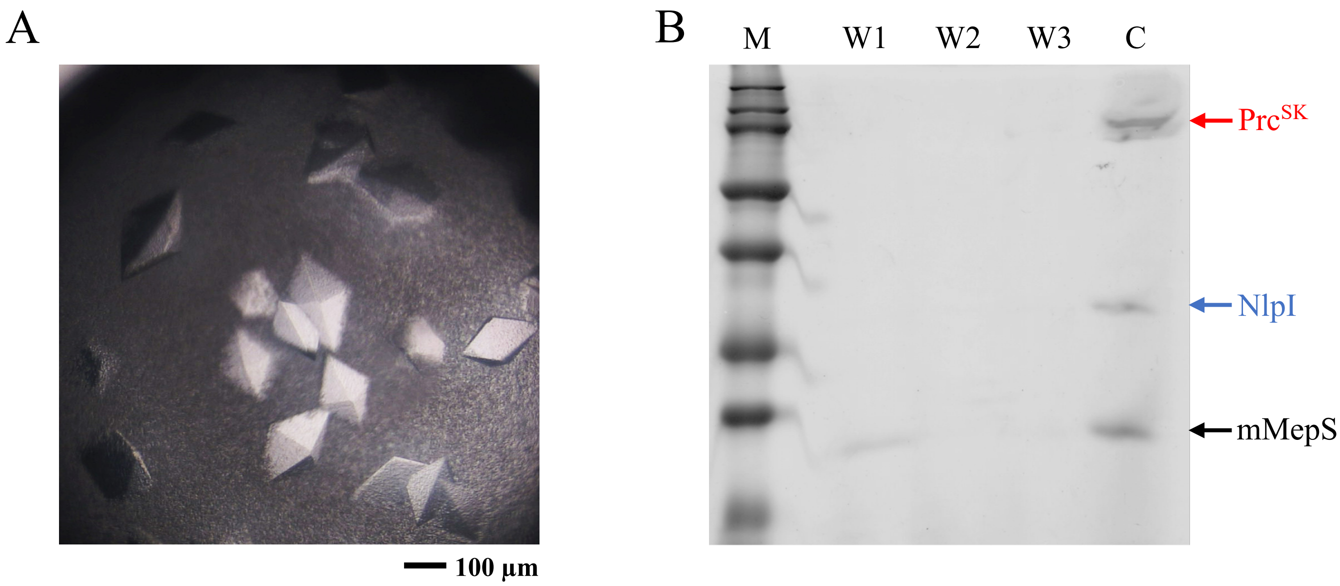

Supplementary Figure 15. Crystallization of Prc^{SK}-NlpI-mMepS complex. (A) The crystals of the Prc^{SK}-NlpI-mMepS complex were generated using the hanging-drop vapor-diffusion method in 24-well plates. Specifically, 1 µl of protein solution was mixed with 1 µl of reservoir solution to initiate crystal formation. (B) the complex was analyzed using SDS-PAGE, with the positions of Prc^{SK}, NlpI, and mMepS indicated by arrowheads in red, blue, and black, respectively.

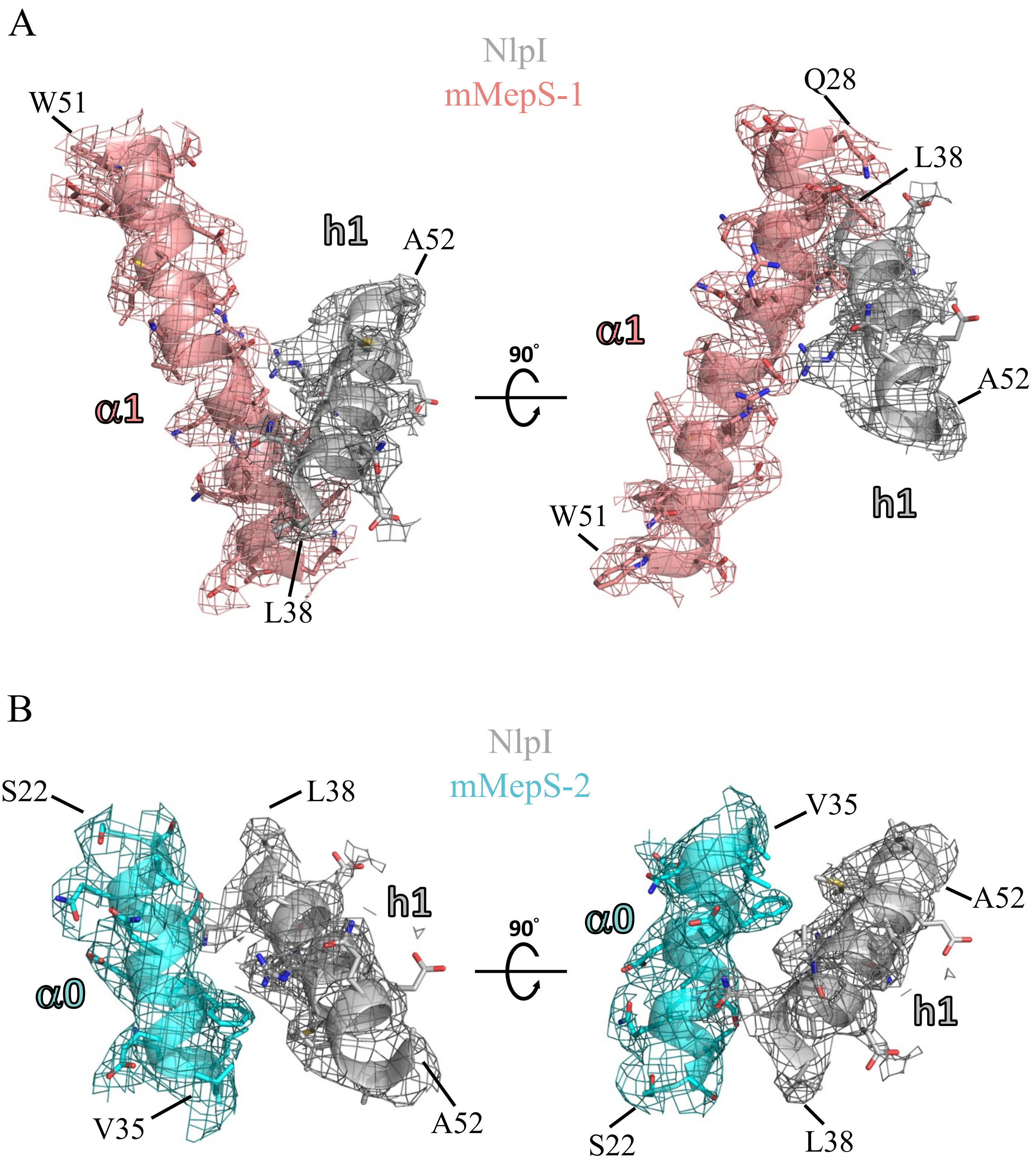

Supplementary Figure 16. The electron density maps for the primary interaction sites between NlpI and two MepS molecules in the Prc^{SK}-NlpI-MepS complex. The spatial orientations of the side-chains of h1 (NlpI, depicted in grey), α1 (mMepS-1, depicted in salmon) and α0 (mMepS-2, depicted in cyan) are easily distinguishable for evaluation.

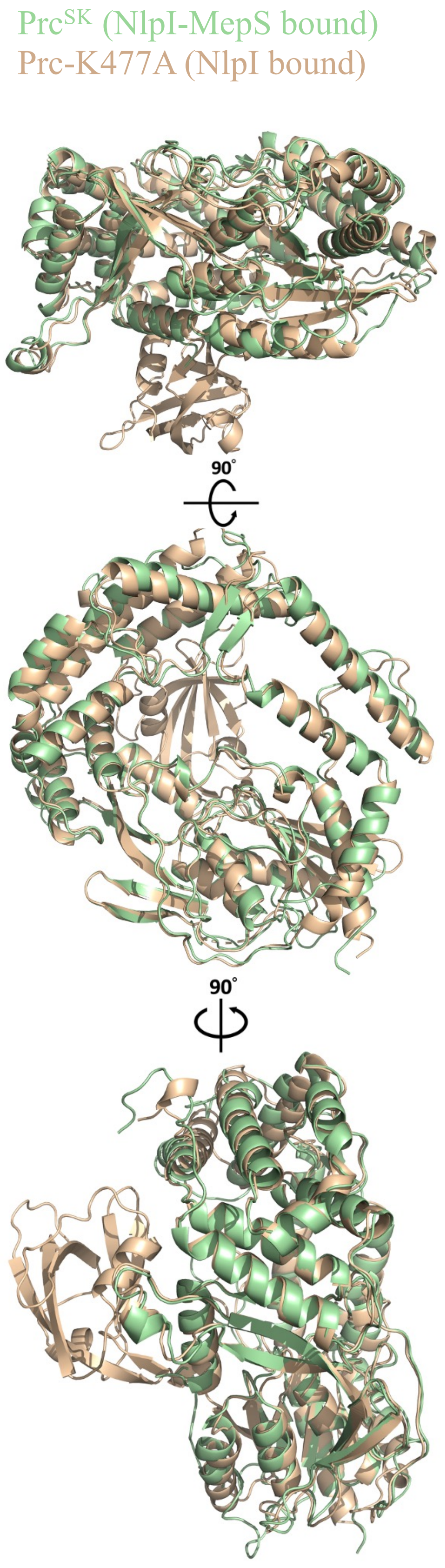

Supplementary Figure 17. Structural comparison of Prc in different protein-bound states. The conformation of Prc^{SK} in the NlpI-mMepS bound state also exhibits a bowl-shaped structure, resembling the structural characteristics of Prc-K477A in the NlpI-bound state.

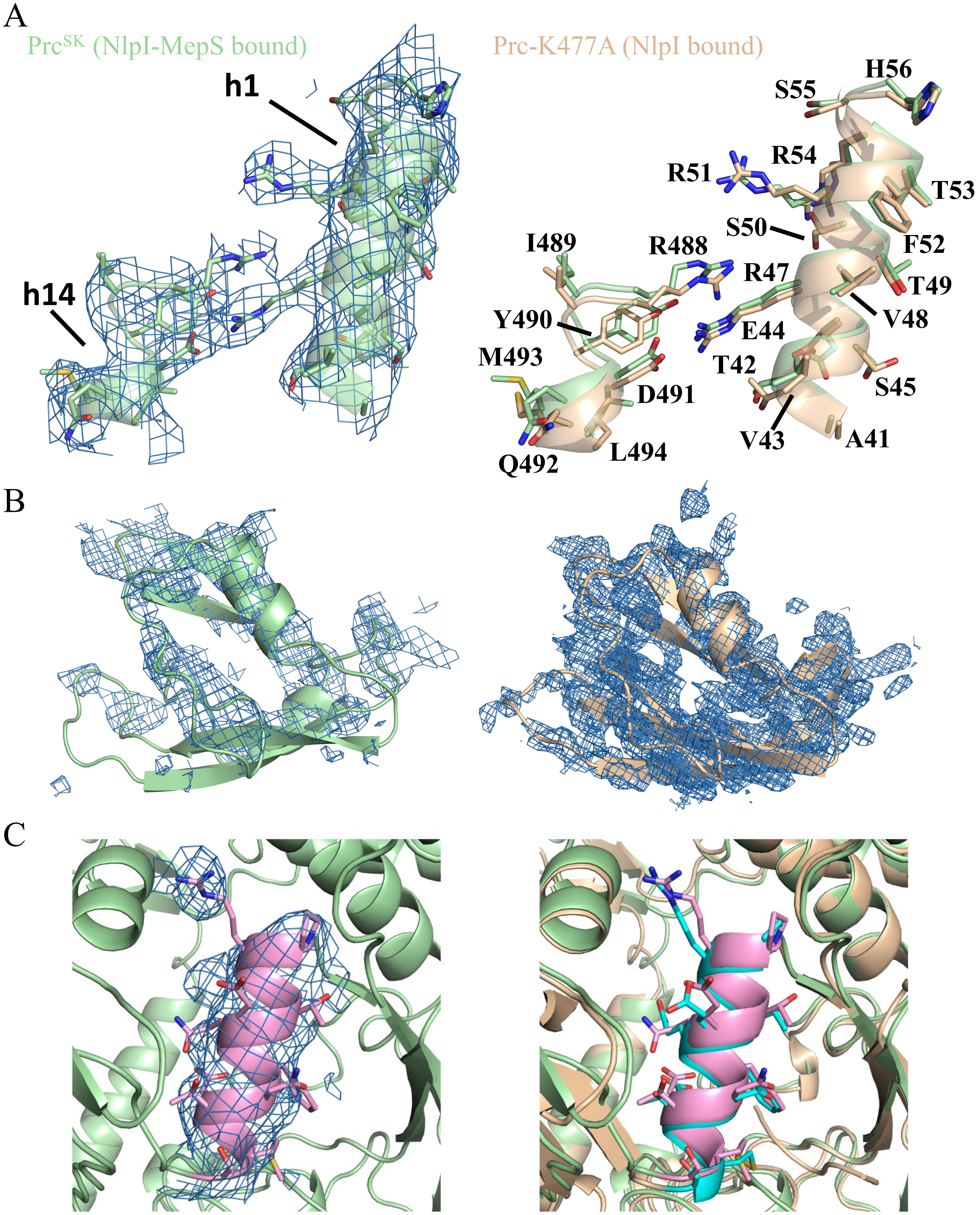

Supplementary Figure 18. The local electron density maps for Prc^{SK} in the NlpI-mMepS-bound state. (A) The electron density map of the NlpI-interaction domain (NID), encompassing h1 and h14, indicates that the NID of NlpI-mMepS-bound Prc^{SK} can be accurately interpreted and bears resemblance to that of NlpI-bound Prc-K477A. (B) The electron density maps for the PDZ domain reveal that the PDZ domain of Prc^{SK} exhibits poor density and is insufficient for definition in the Prc^{SK}-NlpI-mMepS complex. (C) The electron density maps for h9 demonstrate the nearly identical positioning of h9 in the Prc^{SK}-NlpI-mMepS complex compared to that of NlpI-bound Prc-K477A, indicating an active conformation in our structure. The locations of h9 in the Prc^{SK}-NlpI-mMepS and NlpI-Prc-K477A complexes are highlighted in pink and cyan, respectively.

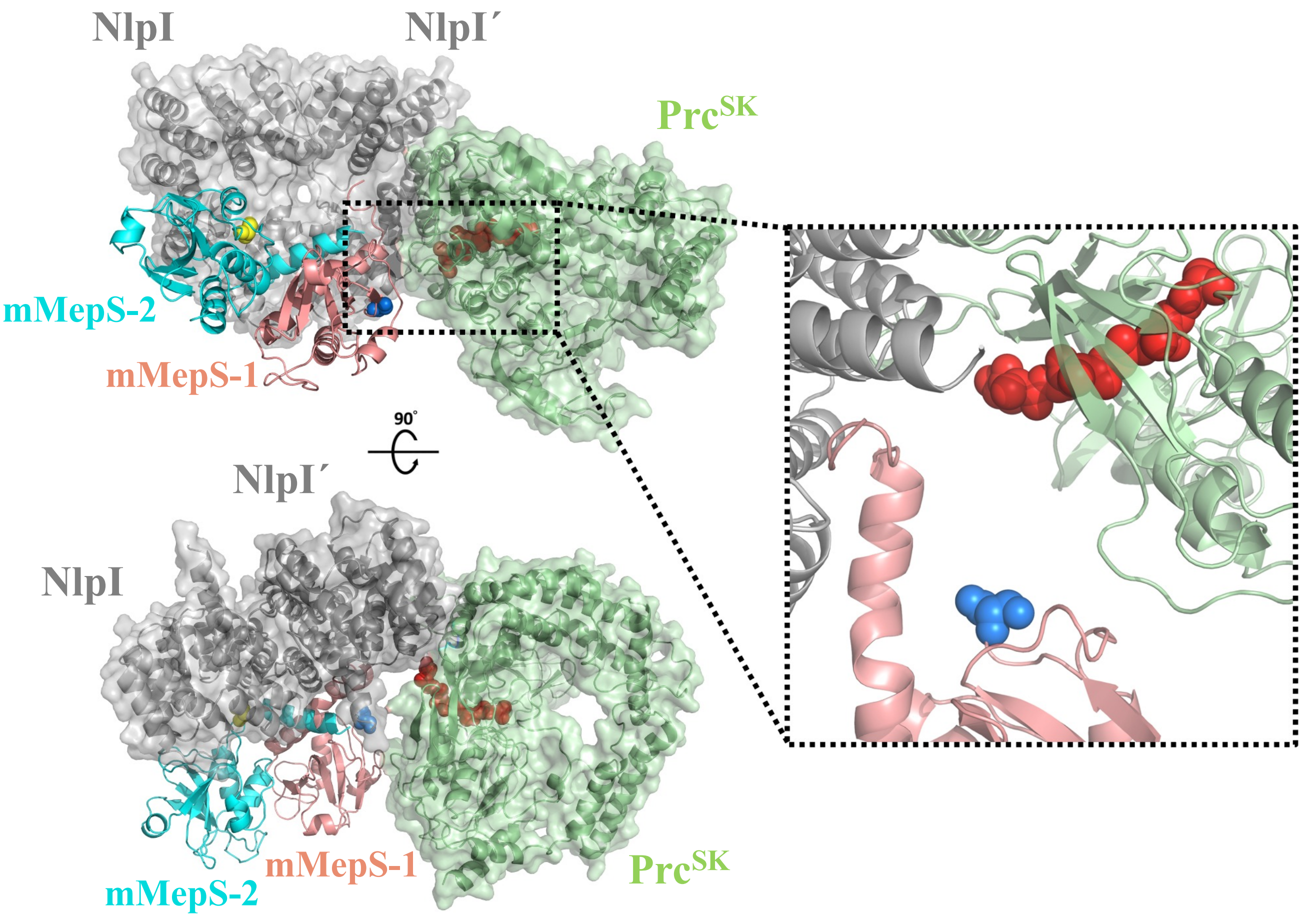

Supplementary Figure 19. The close proximity of the C-terminal of mMepS to the substrate-binding groove of Prc^{SK}. The electron density map surrounding the PDZ domain exhibits low resolution, making it difficult to discern specific details. As a result, our analysis is limited to measuring the distance between the C-terminus of mMepS and the active site of Prc^{SK}. To visually represent this, the bound peptide is represented in red spheres, while the C-terminus of mMepS is depicted in blue spheres to differentiate between the two.

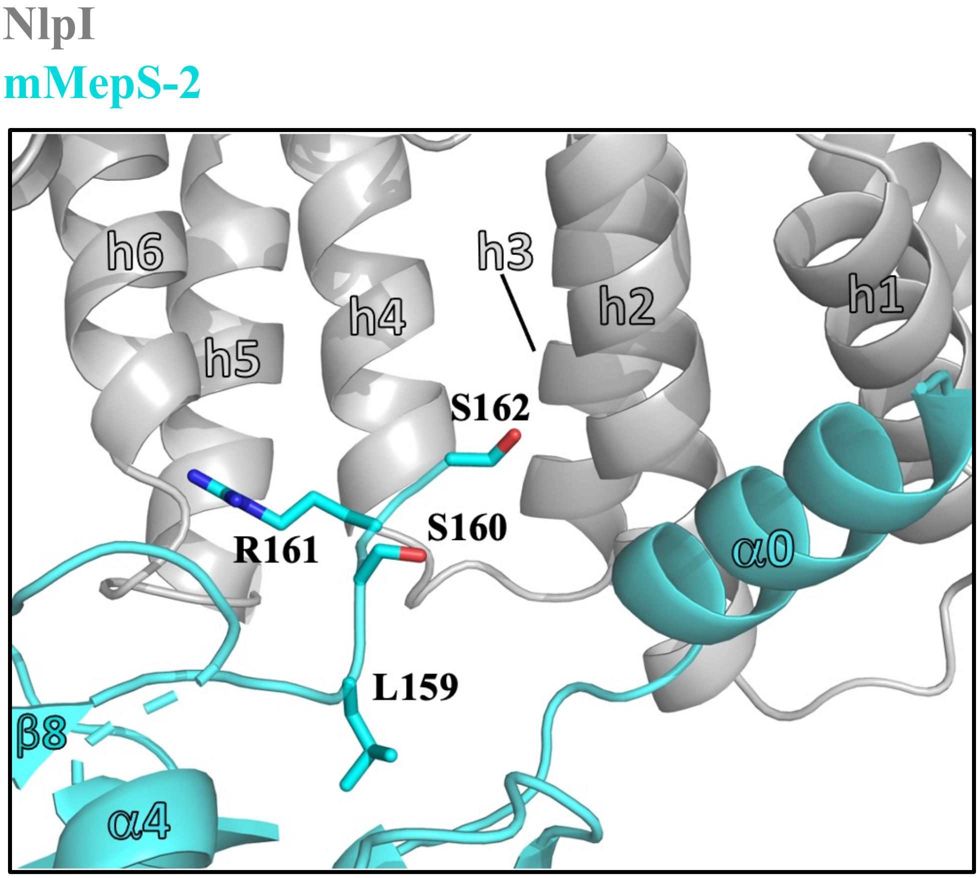

Supplementary Figure 20. A zoom-in view of the C-terminus of mMepS-2. The concave surface (h2-h6) of NlpI encircles the C-terminus of mMepS-2, indicating that Prc may not initially target mMepS-2 for degradation. The specific C-terminal residues (L159, S160, R161 and S162) of mMepS-2 have been identified and labeled.

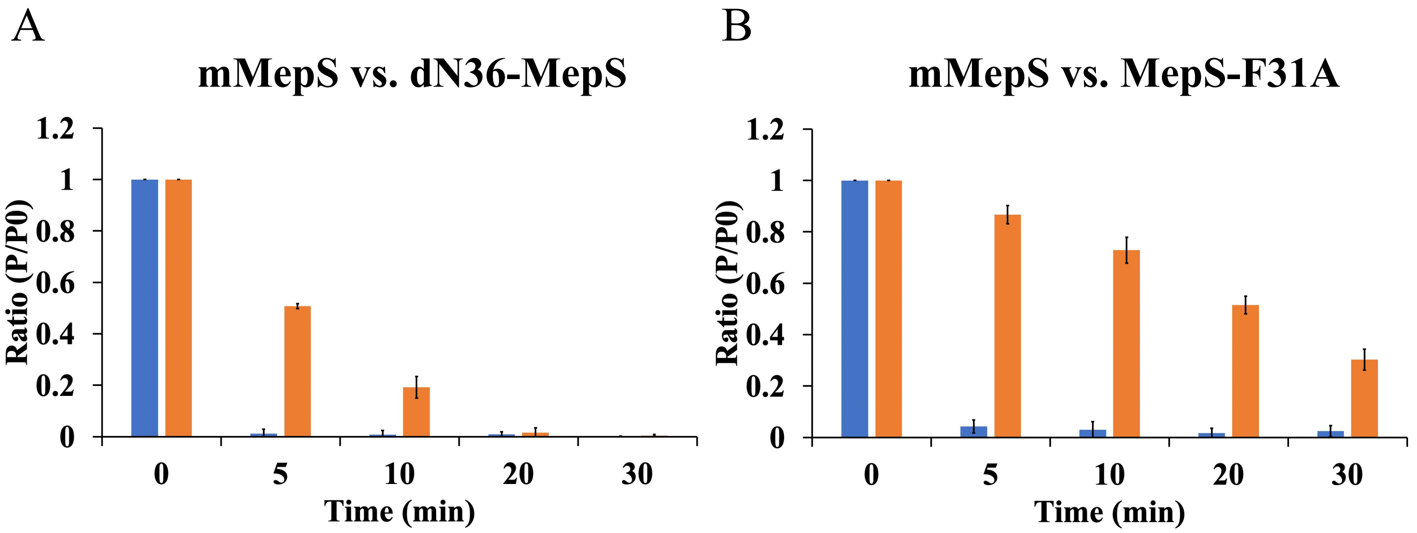

Supplementary Figure 21. The effectiveness of the Prc-NlpI proteolytic system in cleaving various MepS variants was evaluated. (A and B) The residual substrate levels were quantified according to the procedures described in the Materials and Methods section. The blue bars represent the remaining intensity of mMepS, while the orange bars in the left and right charts represent the remaining intensity of dN36-MepS and MepS-F31A, respectively. The bar plots, along with error bars, depict the mean values +/− standard deviation from three independent measurements. The source data is included in a separate Source Data file.

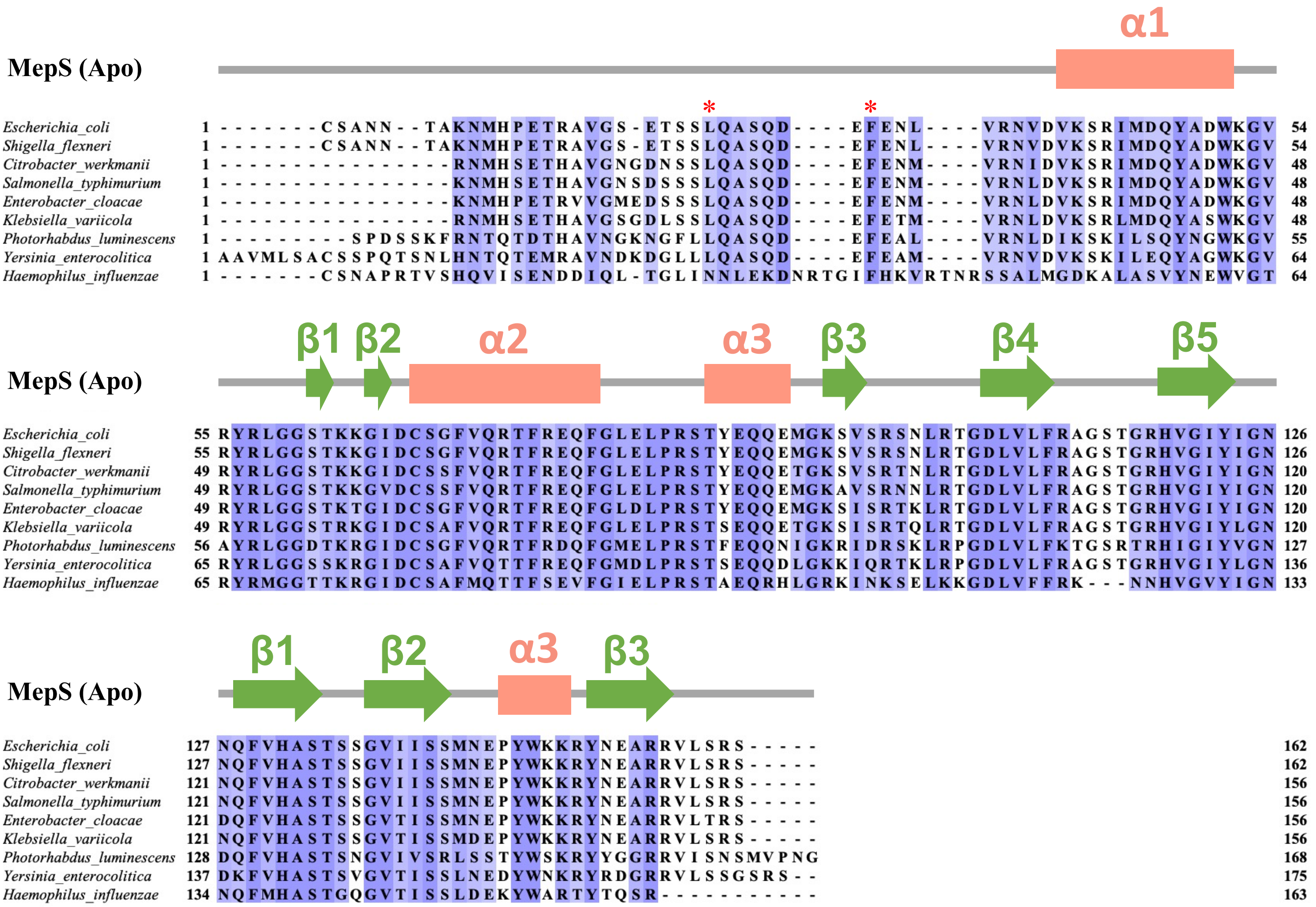

Supplementary Figure 22. A multiple sequence alignment comparing the mature form of MepS peptidase from *E. coli* with chromosomally encoded MepS orthologs using Clustal Omega. The diagram at the top illustrates the assigned secondary structure of MepS, determined using the program STRIDE. Helices are represented by red rectangles and strands by green arrows. UniProt IDs for MepS from various organisms are listed, including *Escherichia coli* (P0AFV4), *Shigella flexneri* (P0AFV7), *Citrobacter werkmanii* (A0A5P2MBM9), *Salmonella typhimurium* (A0A0F7JB69), *Enterobacter cloacae* (A0A0F1D9A5), *Klebsiella variicola* (A0A087FPX5), *Photorhabdus luminescens* (A0A1G5RCK8), *Yersinia enterocolitica* (A0A0E1NJC7), and *Haemophilus influenzae* (P45296). Conserved residues are indicated in purple, and two key hydrophobic residues (L24 and F31) in the N-terminal region of MepS, known for their role in NlpI binding, are denoted by red asterisks.

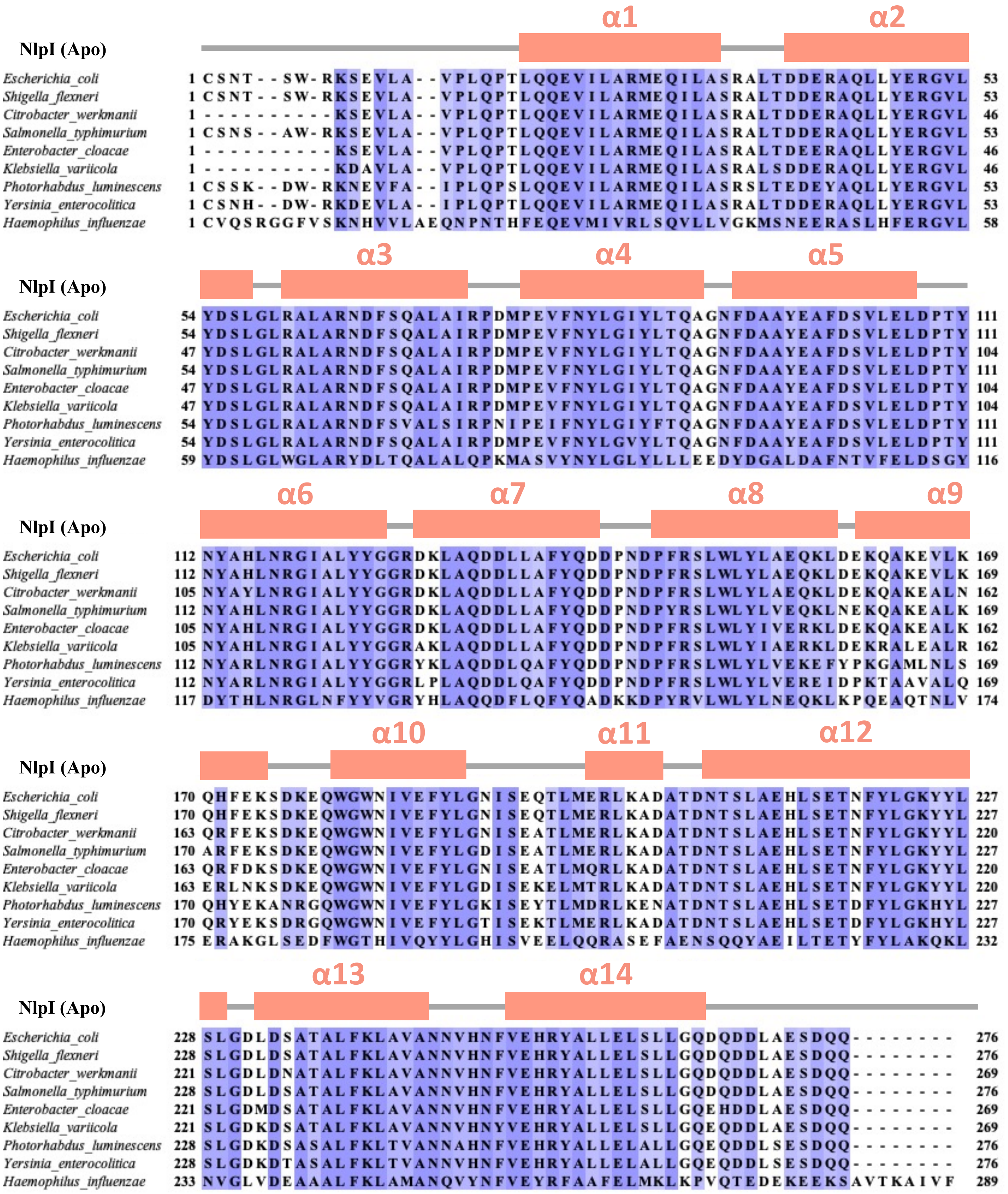

Supplementary Figure 23. A multiple sequence alignment comparing the NlpI from *E. coli* with chromosomally encoded NlpI orthologs using Clustal Omega. The schematic at the top illustrates the assigned secondary structure of NlpI, using the program STRIDE. Helices are represented by red rectangles. UniProt IDs for NlpI from various organisms are listed, including *Escherichia coli* (P0AFB1), *Shigella flexneri* (P0AFB4), *Citrobacter werkmanii* (A0A5P2MHC8), *Salmonella typhimurium* (Q7CPQ1), *Enterobacter cloacae* (A0A156X5X7), *Klebsiella variicola* (A0A087FVV7), *Photorhabdus luminescens* (A0A1G5QQK4), *Yersinia enterocolitica* (A0A7U0AVN1), and *Haemophilus influenzae* (P44585). Conserved residues are highlighted in purple.

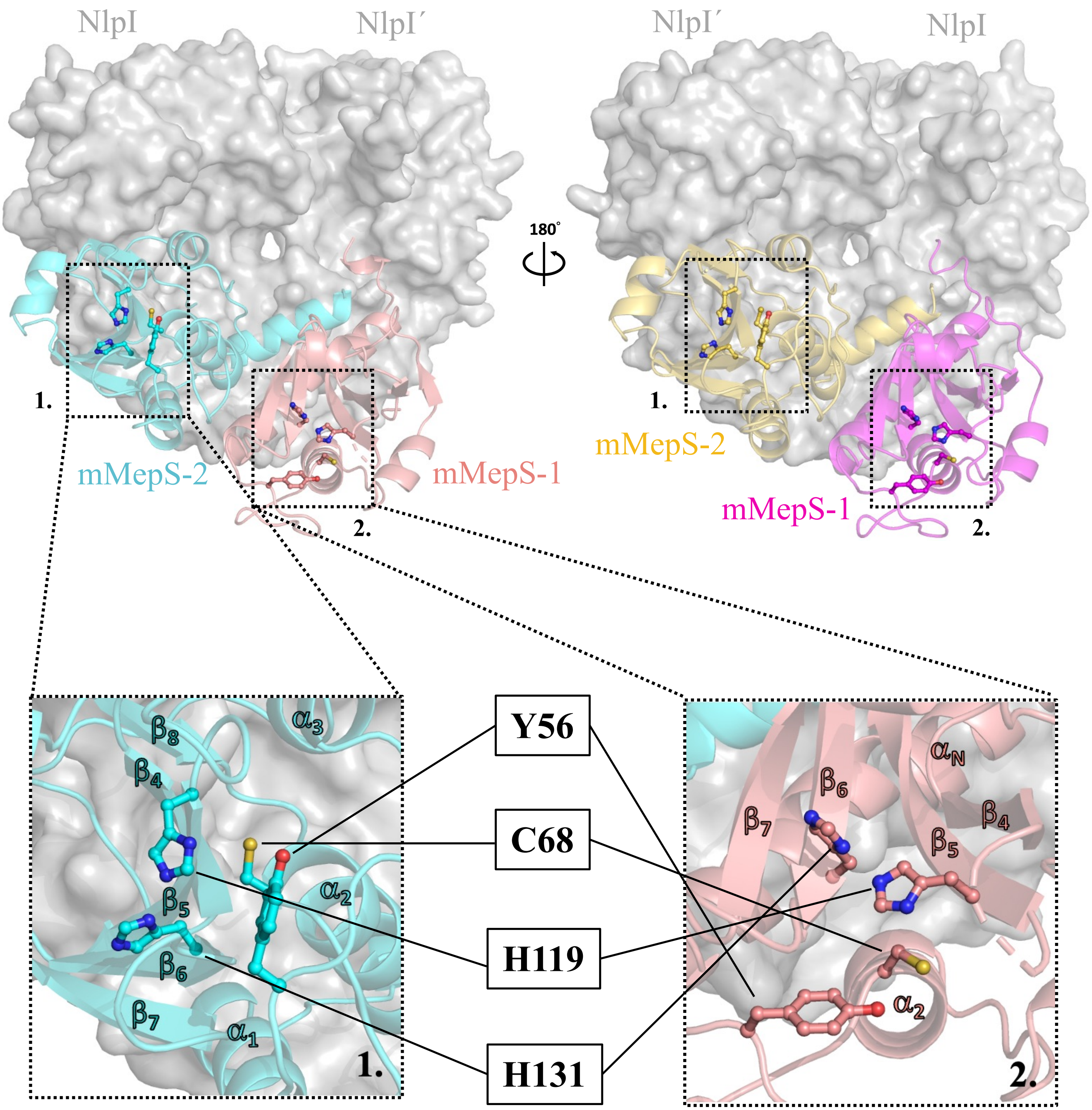

Supplementary Figure 24. The solvent-exposed active-site residues in the NlpI-mMepS complex. Residues believed to be part of the active sites are depicted in the center and visualized using a ball-and-stick model. The arrangement of residues in the active site is remarkably consistent across all four adaptor-bound mMepS.

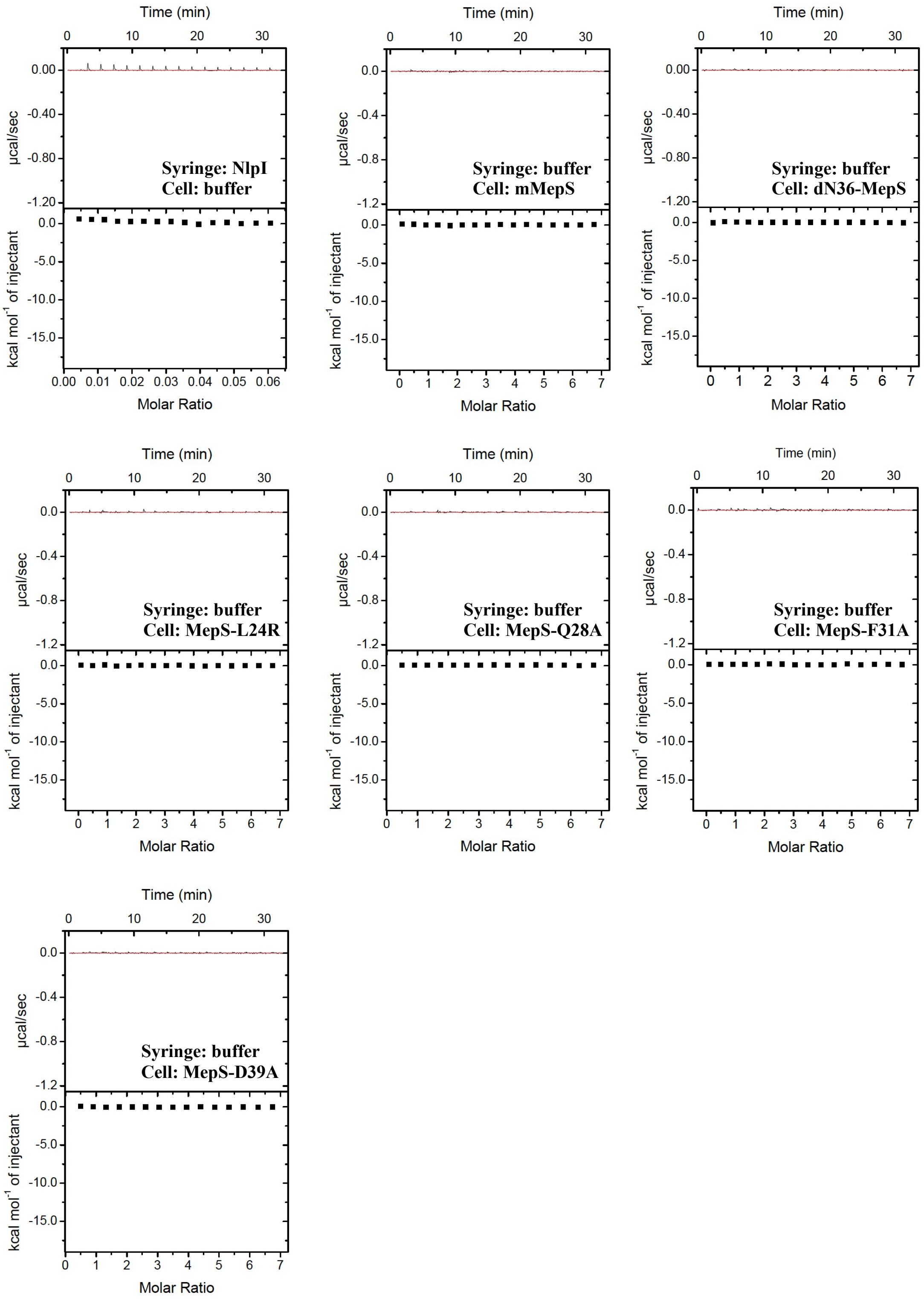

Supplementary Figure 25. ITC control experiments were performed. The precise matching of the buffer in the sample cell and syringe is crucial for accurate titrations. In the process, the titration of the buffer with the protein resulted in minimal heat of dilution.

Supplementary Table 1. Data collection and refinement statistics.

	NlpI-mMepS complex	Prc^{SK}-NlpI-mMepS complex
PDB code	8XUP	8XUD
**Data collection**		
Wavelength (Å)	1.00	1.00
Space group	C121	$P4_32_12$
Cell dimensions		
a, b, c (Å)	197.19, 99.44, 196.24	230.41, 230.41, 182.91
α, β, γ (°)	90, 104.45, 90	90, 90, 90
Resolution (Å)	29.7-2.8 (2.9-2.8)	29.91-3.5 (3.62-3.5)
Unique reflections	90404 (8940)	61469 (6097)
Redundancy	3.8 (3.3)	7.6 (6.8)
Completeness (%)	99.3 (98.8)	97.4 (99.1)
$CC_{1/2}$	0.959 (0.825)	0.951 (0.801)
$I/\sigma(I)$	15.4 (1.8)	19.8 (1.9)
R_{merge}	0.063 (0.382)	0.123 (1.069)
**Refinement**		
R_{work} / R_{free}	0.171 / 0.221	0.239 / 0.281
No. of non-H atoms	18080	19377
Protein	17736	19307
Ion	0	70
Water	344	0
Average *B*-factor		
Protein	64.24	182.23
Ion	-	179.97
Water	45.84	-
R.m.s. deviations		
Bond lengths (Å)	0.008	0.002
Bond angles (°)	1.391	0.63
Ramachandran		
Favored (%)	95.7	95.4
Outliers (%)	0.1	0

*Values of the highest resolution shell are shown in parentheses.

Supplementary Table 2. Thermodynamic parameters determined by ITC.

Cell	Syringe	Expt. number	K_D (10^{-6}/M)	ΔG (kcal mol^{-1})	-TΔS (kcal mol^{-1})	ΔH (kcal mol^{-1})
mMepS	NlpI	No.1	0.25 ± 0.02	-8.85	9.48	-18.33 ± 0.13
		No.2	0.22 ± 0.03	-8.93	9.46	-18.39 ± 0.17
		No.3	0.25 ± 0.03	-8.85	10.88	-19.73 ± 0.16
		Average	0.24 ± 0.02	-8.88 ± 0.04	9.94 ± 0.81	-18.82 ± 0.79
dN36	NlpI	No.1-3	N.D.	N.D.	N.D.	N.D.
N53	NlpI	No.1	1.19 ± 0.10	-7.94	6.52	-14.46 ± 0.24
		No.2	1.56 ± 0.11	-7.79	9.82	-17.61 ± 0.25
		No.3	1.59 ± 0.15	-7.77	6.57	-14.34 ± 0.37
		Average	1.45 ± 0.22	-7.83 ± 0.09	7.64 ± 1.89	-15.47 ± 1.85
N39	NlpI	No.1	2.65 ± 0.21	-7.48	8.01	-15.49 ± 0.32
		No.2	2.55 ± 0.25	-7.50	8.24	-15.74 ± 0.51
		No.3	2.84 ± 0.26	-7.44	7.97	-15.41 ± 0.40
		Average	2.68 ± 0.15	-7.47 ± 0.03	8.07 ± 0.15	-15.55 ± 0.17
L24R	NlpI	No.1	6.21 ± 0.79	-6.98	5.44	-12.42 ± 0.62
		No.2	6.80 ± 0.76	-6.93	5.16	-12.09 ± 0.57
		No.3	6.94 ± 0.66	-6.92	5.91	-12.83 ± 0.52
		Average	6.65 ± 0.39	-6.94 ± 0.03	5.50 ± 0.38	-12.45 ± 0.37
Q28A	NlpI	No.1	1.91 ± 0.28	-7.67	3.03	-10.7 ± 0.30
		No.2	1.77 ± 0.19	-7.71	3.09	-10.8 ± 0.26
		No.3	1.88 ± 0.13	-7.68	3.82	-11.5 ± 0.21
		Average	1.85 ± 0.07	-7.68 ± 0.02	3.31 ± 0.44	-11 ± 0.44
F31A	NlpI	No.1-3	N.D.	N.D.	N.D.	N.D.
D39A	NlpI	No.1	0.28 ± 0.05	-8.79	7.69	-16.48 ± 0.26
		No.2	0.39 ± 0.04	-8.59	8.63	-17.22 ± 0.20
		No.3	0.50 ± 0.08	-8.45	8.83	-17.28 ± 0.24
		Average	0.39 ± 0.11	-8.61 ± 0.17	8.38 ± 0.61	-16.99 ± 0.45

K_D, dissociation constant; ΔG, free energy of binding; ΔH, enthalpy of binding; ΔS, entropy of binding; N.D., not determined.

Supplementary Note 1- Detailed quantitative analysis of SAXS

- Composition determination with SEC-SAXS

We performed size-exclusion-column small-angle X-ray scattering (SEC-SAXS) for the protein complex solution. As demonstrated in previous studies[1,2], SEC-SAXS could determine the composition of the protein complex Prc^{SK}-NlpI-mMepS comprising 2 of Prc^{SK}, 2 of NlpI, and 4 of mMepS (referred as 2P2N4M), through a combined analysis of the zero-angle scattering intensity of SAXS I_0 and UV-absorption intensity I_{UV}, which were concomitantly measured in a same sample elution. The detailed quantitative analysis shown below is consistent with the complex composition of 2P:2N:4M for the hetero-octameric complex.

The composition of the Prc^{SK}-NlpI-mMepS complex 2P2N4M was determined from the SAXS zero-angle scattering intensity I_0 and the sample concentration quantified from UV-vis absorption, as elaborated in the following description.

The complex composition from the X-ray zero-angle scattering intensity I_0 value of the protein solution was calculated using the equation (1).

$$I_0 = \frac{C\Delta\rho^2 v^2 M_w}{N_A} \quad (1)$$

Here, I_0 is determined by the protein concentration C (mass per unit volume), protein molecular weight Mw, the partial specific volume of the protein v, the scattering contrast $\Delta\rho$, and the Avogadro's number N_A, as detailed in our previous report.[1] The scattering contrast $\Delta\rho = \rho_p - \rho_b$ is the difference of the scattering-length-density (SLD) of the protein ρ_p and salt buffer ρ_b, which could be deduced from the respective electron numbers and volumes of the protein and buffer.[1-2] On the basis of (1), the concentration-normalized SAXS intensity I_0/C of 2P2N4M can be deduced in the following

$$I_0/C = \frac{\Delta\rho^2 v^2 M_w}{N_A} = \frac{10^{-3}\times\left(2.3459*10^{10}\ cm^{-2}\right)^2\times(0.7582\ {}^{cm^3}/_{g})^2\times(289630\ {}^{g}/_{mol})}{6.022*10^{23}\ mol^{-1}} = 0.1522\ \frac{(cm^{-1})}{(mg/ml)} \quad (2)$$

with
C the sample concentration in units of [mg/ml] or [10^{-3} g/cm^3],
N_A the Avogadro's number = 6.022×10^{23} mol^{-1}, and
$\rho_b = 9.6110\times10^{10}$ cm^{-2} for the scattering length density of buffer with 0.2 M Na/K phosphate at 283 k.
$\rho_p = 11.9568\times10^{10}$ cm^{-2} is the scattering length density of the protein complex 2P2N4M, which is deducted from the total electrons and total dry volume $V = 365237$ Å^3 of the complex 2P2N4M (calculated on the basis of a previous report[3]).
$\Delta\rho = \rho_p - \rho_b = 2.3459\times10^{10}$ cm^{-2} is the scattering contrast, with
$v = V/M_W = 0.7582$ cm^3/g for the partial specific volume defined by V and the molar mass
$M_W = 290082$ Da of the complex 2P2N4M.

Supplementary Note 1- Continued

With the SEC-SAXS of the TPS 13 BioSAXS beamline[4,5], the SAXS I_0 and UV–Vis absorption measurements at the same sample position; the sample maximum concentration of the elution peak can be determined by the detected UV–Vis optical density corresponding SAXS I_0 measured. The protein concentration C (mass per unit volume) in our sample can be calculated using equation (3), based on Beer's Law.

$$I_{UV} = C\varepsilon L. \quad (3)$$

Here, the protein concentration C is determined by the extinction coefficient ε and the path length of the sample in the flow cell of an optical path length L. The extinction coefficient for Prc^{SK}-NlpI-mMepS, which has a composition of 2:2:4, is 1008 mAU $cm^{-1}(mg/ml)^{-1}$. The calibrated light path of the online UV-vis detection L = 0.201 cm. The measured elution peak UV absorbance of the PrcSK-NlpI-mMepS complex is 280 mAU. Based on (3), the peak sample concentration

$$C = I_{UV}/\varepsilon L = \frac{280\ mAU}{1008\ mAU\cdot cm^{-1}\cdot(mg/ml)^{-1}\times 0.201\ cm} = 1.389\ mg/ml \quad (4)$$

Combined (2) and (4), we obtain the elution peak SAXS I_0 = 0.211 cm^{-1} for 2P2N4M. This I_0 value calculated from the composition of 2:2:4 of the complex 2P2N4M aligns very well with the measured SAXS I_0 value of 0.213 ± 0.001 cm^{-1}. The consistent result provides strong support to the composition of 2:2:4 of the complex 2P2N4M with a confidence level of over 90% (estimated from the small difference in the calculated and measured I_0 values).

Supplementary References

1. Shih, O. et al., Membrane charging and swelling upon calcium adsorption as revealed by phospholipid nanodiscs, *J. Phys. Chem. Lett.* **9**, 4287–4293 (2018).
2. Shih, O. et al., Solution structure of bilayer membrane-embedded proton-translocating pyrophosphatase revealed via small-angle X-ray scattering, *Mater. Chem. Phys.* **308**, 128253 (2023).
3. Jacrot, B., The study of biological structures by neutron scattering from solution, *Rep. Prog. Phys.* **39**, 911-953 (1976)
4. Shih, O. et al. Performance of the new biological small- and wide-angle X-ray scattering beamline 13A at the Taiwan Photon Source. *J. Appl Crystallogr* **55**, 340–352 (2022).
5. Liu, D. G. et al. Optical design and performance of the biological small-angle X-ray scattering beamline at the Taiwan Photon Source. *J. Synchrotron Radiat.* **28**, 1954–1965 (2021).
