# Supplementary material for: Structural basis for recruitment of peptidoglycan endopeptidase MepS by lipoprotein NlpI
Source: Nat Commun. 2024 Jun 27;15:5461. doi: 10.1038/s41467-024-49552-y (PMC11211486; doi:10.1038/s41467-024-49552-y)
Supplement: Supplementary file 2 — Reporting Summary [file 41467_2024_49552_MOESM2_ESM.pdf]

nature portfolio

Corresponding author(s): Shiou-Ru Tzeng

Last updated by author(s): Jun 3, 2024

Reporting Summary

Nature Portfolio wishes to improve the reproducibility of the work that we publish. This form provides structure for consistency and transparency in reporting. For further information on Nature Portfolio policies, see our Editorial Policies and the Editorial Policy Checklist.

Statistics

For all statistical analyses, confirm that the following items are present in the figure legend, table legend, main text, or Methods section.

n/a	Confirmed	
☐	☒	The exact sample size (*n*) for each experimental group/condition, given as a discrete number and unit of measurement
☐	☒	A statement on whether measurements were taken from distinct samples or whether the same sample was measured repeatedly
☒	☐	The statistical test(s) used AND whether they are one- or two-sided<br>*Only common tests should be described solely by name; describe more complex techniques in the Methods section.*
☒	☐	A description of all covariates tested
☒	☐	A description of any assumptions or corrections, such as tests of normality and adjustment for multiple comparisons
☐	☒	A full description of the statistical parameters including central tendency (e.g. means) or other basic estimates (e.g. regression coefficient) AND variation (e.g. standard deviation) or associated estimates of uncertainty (e.g. confidence intervals)
☒	☐	For null hypothesis testing, the test statistic (e.g. *F*, *t*, *r*) with confidence intervals, effect sizes, degrees of freedom and *P* value noted<br>*Give P values as exact values whenever suitable.*
☒	☐	For Bayesian analysis, information on the choice of priors and Markov chain Monte Carlo settings
☒	☐	For hierarchical and complex designs, identification of the appropriate level for tests and full reporting of outcomes
☒	☐	Estimates of effect sizes (e.g. Cohen's *d*, Pearson's *r*), indicating how they were calculated

Our web collection on statistics for biologists contains articles on many of the points above.

Software and code

Policy information about availability of computer code

Data collection	NMR data were collected using Bruker TopSpin (version 3.2), a standard commercial program.<br>All SEC data were collected with UNICORN (version 5.31).<br>All X-ray data were collected using Blu-Ice (version 5.0).<br>All SAXS data were collected using TPS 13A1 Data Collecting UI (DC-GUI) Ver 2.18 1M/9M.<br>All ITC data were collected using Microcal iTC200 (version 1.26.1)
Data analysis	NMR data were processed with Bruker TopSpin (version 4.0.6) and NMRPipe (version mac).<br>NMR spectra were visualized and analyzed using NMRviewJ (version 9.0.0-b110).<br>NMR resonance assignment were completed using CARA 2 (version 1.9.1.7).<br>All SEC data were analyzed with UNICORN (version 5.31).<br>All collected X-ray data were processed using HKL2000 package (version 722) or XDS (version Jun 30, 2023).<br>All collected SAXS data were processed and evaluated using TPS 13A1 SWAXS data reduction kit (DRK) Ver 4.88.<br>Map and model refinement were processed with COOT 1.0 (version mac) and PHENIX (version 1.18.2).<br>Structure ensembles were colored and labeled for visualization using PyMOL (version 2.5.2).<br>All ITC data were processed and analyzed using Origin 7 (version 7.0552).

For manuscripts utilizing custom algorithms or software that are central to the research but not yet described in published literature, software must be made available to editors and reviewers. We strongly encourage code deposition in a community repository (e.g. GitHub). See the Nature Portfolio guidelines for submitting code & software for further information.

Data

Policy information about availability of data

All manuscripts must include a data availability statement. This statement should provide the following information, where applicable:

- Accession codes, unique identifiers, or web links for publicly available datasets
- A description of any restrictions on data availability
- For clinical datasets or third party data, please ensure that the statement adheres to our policy

The crystal structures of MepS bound to NlpI or NlpI-Prc complex and the coordinates have been deposited at the Protein Data Bank with accession code PDB ID 8XUP and 8XUD. The NMR chemical shifts of full length MepS were deposited at the Biological Magnetic Resonance Bank (BMRB) under BMRB entry ID 51949. Previously reported structures mentioned in our study are under the accession codes in PDB: 1XNF, 2K1G, 7V6S and 5WQL; BMRB entry ID 15603.

Research involving human participants, their data, or biological material

Policy information about studies with human participants or human data. See also policy information about sex, gender (identity/presentation), and sexual orientation and race, ethnicity and racism.

Reporting on sex and gender	N/A.
Reporting on race, ethnicity, or other socially relevant groupings	N/A.
Population characteristics	N/A.
Recruitment	N/A.
Ethics oversight	N/A.

Note that full information on the approval of the study protocol must also be provided in the manuscript.

Field-specific reporting

Please select the one below that is the best fit for your research. If you are not sure, read the appropriate sections before making your selection.

- [x] Life sciences
- [ ] Behavioural & social sciences
- [ ] Ecological, evolutionary & environmental sciences

For a reference copy of the document with all sections, see nature.com/documents/nr-reporting-summary-flat.pdf

Life sciences study design

All studies must disclose on these points even when the disclosure is negative.

Sample size	All experiments were done at least in duplicate, if applicable.
Data exclusions	For Prc-NlpI-MepS complex, the PDZ domains in our main figure were excluded due to poor electron density.
Replication	All experiments were done at least in duplicate, if applicable.
Randomization	Randomization is not needed in this study, as the objective is to investigate specific biological or physiological mechanisms, test the effects of compounds/treatments, or explore fundamental research questions. The focus is on understanding the underlying processes, rather than evaluating the effectiveness of an intervention in a diverse population.
Blinding	Blinding is not needed in this study, as the objective is to investigate specific biological or physiological mechanisms, test the effects of compounds/treatments, or explore fundamental research questions. The focus is on understanding the underlying processes, rather than evaluating the effectiveness of an intervention in a diverse population.

Reporting for specific materials, systems and methods

We require information from authors about some types of materials, experimental systems and methods used in many studies. Here, indicate whether each material, system or method listed is relevant to your study. If you are not sure if a list item applies to your research, read the appropriate section before selecting a response.

Materials & experimental systems

n/a	Involved in the study	
☐	☒	Antibodies
☒	☐	Eukaryotic cell lines
☒	☐	Palaeontology and archaeology
☒	☐	Animals and other organisms
☒	☐	Clinical data
☒	☐	Dual use research of concern
☒	☐	Plants

Methods

n/a	Involved in the study	
☒	☐	ChIP-seq
☒	☐	Flow cytometry
☒	☐	MRI-based neuroimaging

Antibodies

Antibodies used	The antibodies used in this study include: mouse monoclonal OctA-Probe antibody (Santa Cruz, sc-166355; 1:2,000 for IB), rabbit polyclonal anti-RecA antibody (abcam, ab63797; 1:3000 for IB) and mouse monoclonal anti-NlpI antibody, produced from Leadgene Biomedical, Inc (Tainan, Taiwan).
Validation	Mouse monoclonal OctA-Probe antibody: https://www.scbt.com/p/octa-probe-antibody-h-5<br>Rabbit polyclonal anti-RecA antibody: https://www.abcam.com/products/primary-antibodies/reca-antibody-ab63797.html<br>Leadgene Biomedical, Inc: https://www.leadgenebio.com
